# Antiproliferative activity of new pentacyclic triterpene and a saponin from *Gladiolus segetum* Ker-Gawl corms supported by molecular docking study†

**DOI:** 10.1039/d0ra02775h

**Published:** 2020-06-12

**Authors:** Adel M. Abd El-kader, Basma Khalaf Mahmoud, Dina Hajjar, Mamdouh F. A. Mohamed, Alaa M. Hayallah, Usama Ramadan Abdelmohsen

**Affiliations:** Department of Pharmacognosy, Faculty of Pharmacy, Deraya University Minia Egypt usama.ramadan@mu.edu.eg +20-86-2347759; Department of Pharmacognosy, Faculty of Pharmacy, Al-Azhar University Assiut 71524 Egypt; Department of Pharmacognosy, Faculty of Pharmacy, Minia University 61519 Minia Egypt; Department of Biochemistry, Collage of Science, University of Jeddah 80203 Jeddah Saudi Arabia; Department of Pharmaceutical Chemistry, Faculty of Pharmacy, Sohag University 82524 Sohag Egypt; Pharmaceutical Chemistry Department, Faculty of Pharmacy, Deraya University Minia Egypt; Department of Pharmaceutical Organic Chemistry, Faculty of Pharmacy, Assiut University Assiut 71526 Egypt

## Abstract

A new triterpenoidal saponin identified as 3-*O*-[β-d-glucopyranosyl-(1 → 2)-β-d-glucopyranosyl-(1 → 4)-β-d-xylopyranosyl]-2β,3β,16α-trihydroxyolean-12-en-23,28-dioic acid-28-*O*-α-l-rhamnopyranosyl-(1 → 4)-α-l-rhamnopyranosyl-(1 → 2)-β-d-glucopyranosyl-(1 → 2)-α-l-arabinopyranoside 1 together with a new oleanane triterpene identified as 2β,3β,13α,22α-tetrahydroxy olean-23,28-dioic acid 2 and 6 known compounds (3–8) have been isolated from *Gladiolus segetum* Ker-Gawl corms. The structural elucidation of the isolated compounds was confirmed using different chemical and spectroscopic methods, including 1D and 2D NMR experiments as well as HR-ESI-MS. Moreover, the *in vitro* cytotoxic activity of the fractions and that of the isolated compounds 1–8 were investigated against five human cancer cell lines (PC-3, A-549, HePG-2, MCF-7 and HCT-116) using doxorubicin as a reference drug. The results showed that the saponin fraction exhibited potent *in vitro* cytotoxic activity against the five human cancer cell lines, whereas the maximum activity was exhibited against the PC-3 and A-549 cell lines with the IC_50_ values of 1.13 and 1.98 μg mL^−1^, respectively. In addition, compound 1 exhibited potent activity against A-549 and PC-3 with the IC_50_ values of 2.41 μg mL^−1^ and 3.45 μg mL^−1^, respectively. Interestingly, compound 2 showed the maximum activity against PC-3 with an IC_50_ of 2.01 μg mL^−1^. These biological results were in harmony with that of the molecular modeling study, which showed that the cytotoxic activity of compound 2 might occur through the inhibition of the HER-2 enzyme.

## Abstract 2

A new triterpenoidal saponin 1, a new oleanane triterpene 2, and 6 known compounds (3–8) have been isolated from *Gladiolus segetum* Ker-Gawl corms.

## Graphical abstract

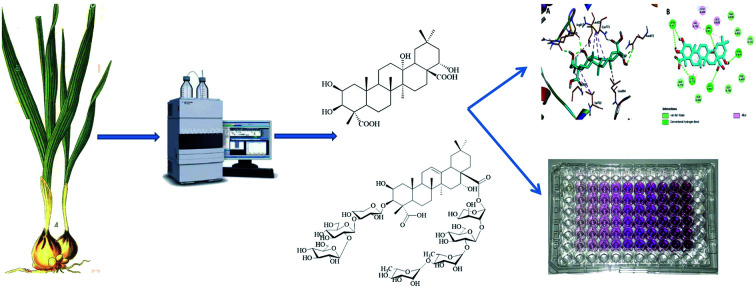

## 1. Introduction

Natural products and their derivatives are of great importance and have been recognized for many years as an important source of therapeutic agents for the treatment of several diseases.^1^ Natural products play a vital role on our earth as the world is decorated with medicinal herbs. These herbs have direct medicinal applications as drug entities and there are also many others that can serve as chemical models or templates for synthesis, semi-synthesis and the design of novel drug candidates for the treatment of human diseases.^2^ The *Gladiolus* genus is one of the attractive medicinal plants belonging to the horticultural family *Iridaceae* and comprises 260 species, which are mainly native to South Africa with several species distributed in Western and Central Europe, the Mediterranean region, and Southwest and Central Asia.^3^ Many *Gladiolus* species corms have been traditionally used as a remedy for dysentery and rheumatic pain,^4^ such as *G*. *gandavensis* corms, which are used to treat pharyngitis, parotitis and lymphadenitis in Chinese folk medicine.^5^ In addition, *G. dalenii* was used in the Kenyan Lake Victoria Basin as a traditional treatment for meningitis, malaria, diarrhoea, ulcers and HIV-related fungal infections. Likewise, the hot water extracts of dried corms are used for the treatment of ulcers.^6^ Moreover, the phytochemical survey of the genus *Gladiolus* showed an interesting wide diversity of chemical constituents, such as anthraquinones,^4,5,7^ flavonoids,^8^ triterpenes,^4^ saponins,^9^ and lignans.^10^ There are several approaches to drug discovery, such as combinatorial chemistry and computer-based molecular modeling design based on natural products,^2^ in addition to drug discovery using natural products, which represents a challenging task for designing new leads. Human epidermal growth factor receptor 2 (HER2) is a member of the epidermal growth factor receptor family having tyrosine kinase activity,^11^ which can regulate cell growth, survival, and differentiation *via* multiple signal transduction pathways and participate in cellular proliferation and differentiation.^12^ Most studies on HER2 have been carried out in breast cancer and its overexpression occurs in approximately 15–30% of breast cancers.^13–15^ It has now been documented that HER2 overexpression also occurs in many forms of cancers, such as ovary, stomach, uterine serous endometrial carcinoma, bladder, lung, colon, uterine cervix, head and neck, and esophagus.^16,17^ Its major role in these tissues is to facilitate excessive/uncontrolled cell growth and tumorigenesis.^18–20^ Therefore, HER2 has been recognized as a promising anticancer target with many reported inhibitors such as Trastuzumab, Lapatinib, Pertuzumab, Neratinib and Afatinib.^11^ Therefore, the present study deals with the isolation and structural elucidation of eight compounds from *Gladiolus segetum* Ker-Gawl corms, which is a herbaceous plant cultivated in Egypt as an ornamental plant and known locally as Seif El-Ghorab.^10^ Moreover, we evaluated the antiproliferative activity of the pure compounds on five different cell lines. Finally, the docking study of the isolated compounds was carried out against human epidermal receptor 2 (HER-2) at the catalytic ligand binding site (PDBID: 5jeb) to help better understand the binding mode and their possible mechanism of action as potential new anticancer agents.

## 2. Results and discussion

**Fig. 1:**
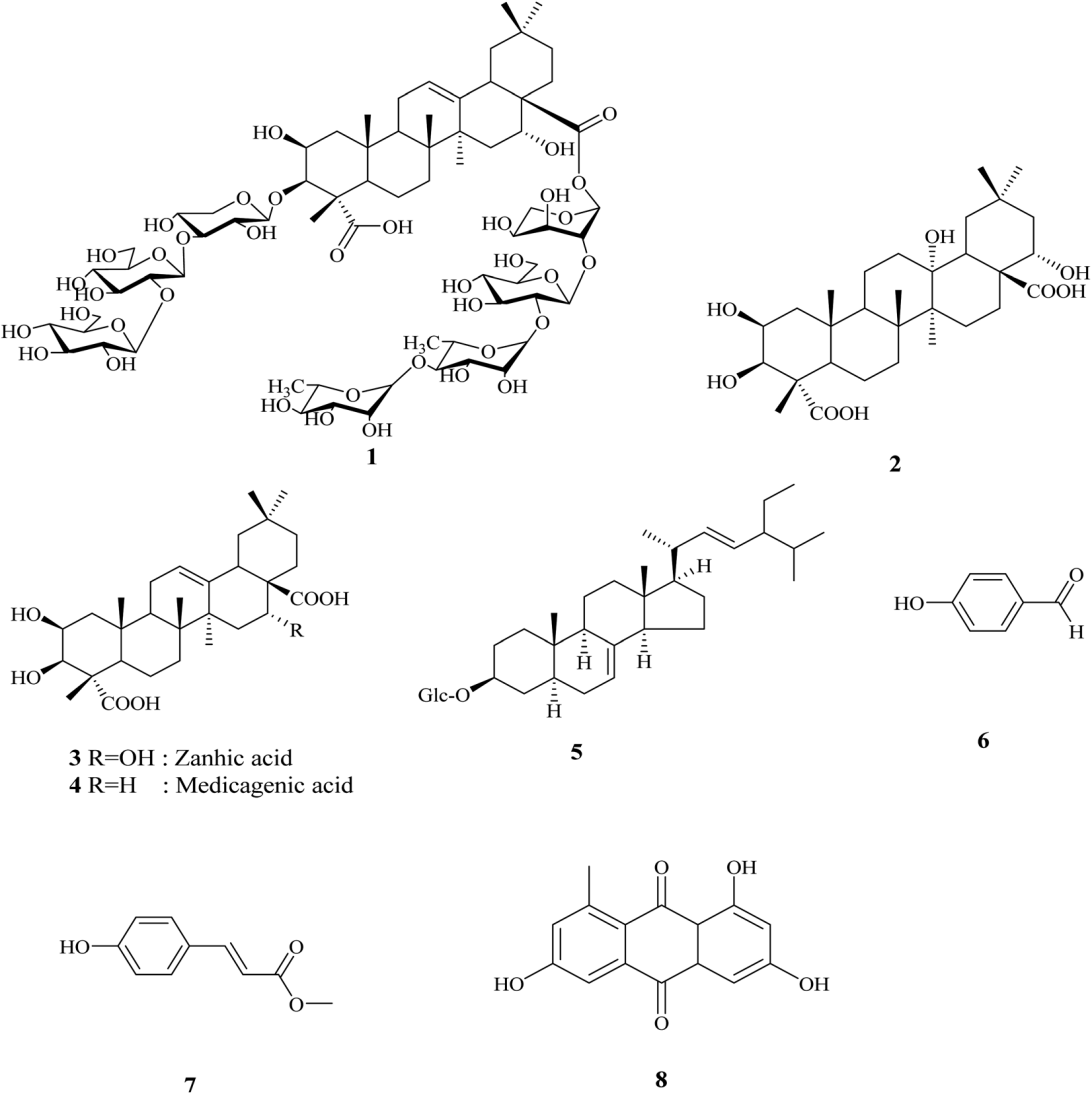
Structures of the isolated compounds from *Gladiolus segetum* Ker-Gawl corms.

The crude extract of *Gladiolus segetum* Ker-Gawl corms was chromatographed using various chromatographic techniques, such as silica gel, Sephadex LH-20, RP_18_ column chromatography, and the final purification on HPLC led to the identification of two new compounds, in addition to six known compounds, as shown in Fig. 1.

### 2.1. Structure elucidation of the new compounds

Compound 1 was obtained as a white amorphous powder. Its molecular formula was established as C_70_H_112_O_38_ based on the positive ion mode HR-ESI-MS. The ^1^H-NMR spectrum of compound 1 revealed the presence of six characteristic singlet signals for six methyl groups at *δ*_H_ 0.66, 0.83, 0.90, 1.14, 1.18, and 1.28 (each 3H, s), in addition to an olefinic proton at *δ*_H_ 5.21, suggesting the oleanane triterpene skeleton of the aglycon. This was further confirmed from the ^13^C-NMR spectra that revealed the presence of six tertiary carbon signals, in addition to one olefinic carbon at *δ*_C_ 121.83 and one quaternary carbon signal at *δ*_C_ 143.26, and two carbonyl carbon signals at *δ*_C_ 180.73 (C-23) and 175.34 (C-28). Therefore, the structure of the triterpenoid moiety was obtained principally from the correlations observed in the HSQC and HMBC spectra, and confirmed to be that of 2β,3β,16α-trihydroxyolean-12-en-23,28-dioic acid (zanhic acid) by comparison of the NMR data (Table 1) with those from the literature.^21,22^

**Table 1:** ^1^H and ^13^C spectral data of compound 1 (600 MHz and 150 MHz, respectively, DMSO-*d*_*6*_)

No.	Aglycone		3-*O*-sugar units			28-*O*-sugar units		
	*δ*_H_ (m, *J* in Hz)	*δ*_C_		*δ*_H_ (m, *J* in Hz)	*δ*_C_		*δ*_H_ (m, *J* in Hz)	*δ*_C_
1	1.14, 1.87 (m)	43.66	**Xyl**			**Ara**		
2	3.93 (br s)	68.63	**1′**	4.25 (d, 7.0)	**105.76** a	**1′′′′**	5.19 (s)	**93.27** a
3	3.81 (br s)	80.40	**2′**	3.30 (m)	75.21	**2′′′′**	3.33 (m)	**75.70** a
4	—	51.42	**3′**	4.05 (m)	**84.20** a	**3′′′′**	3.36 (m)	71.33
5	1.40 (br s)	50.21	**4′**	3.58 (m)	70.68	**4′′′′**	3.59 (m)	67.38
6	1.08, 1.17 (m)	18.15	**5′**	3.35 (m), 3.65 (m)	67.70	**5′′′′**	3.34, (m) 3.64 (m)	64.97
7	1.25, 1.58 (m)	34.83						
8	—	39.01	**Glc I**	4.19 (d, 7.21)		**GlcIII**		
9	1.44 (br s)	47.86	**1′′**	3.78 (m)	**103.20** a	**1′′′′′**	4.21 (d, 7.34)	**103.18** a
10	—	36.76	**2′′**	3.56 (m)	**81.60** a	**2′′′′′**	3.79 (m)	**81.95** a
11	0.85, 1.81 (m)	23.13	**3′′**	3.62 (m)	78.99	**3′′′′′**	3.42 (m)	79.20
12	5.21 (br s)	121.83	**4′′**	3.18 (m)	70.62	**4′′′′′**	3.37 (m)	71.25
13	—	143.26	**5′′**	3.43 (m), 3.69 (m)	76.10	**5′′′′′**	3.38 (m)	76.87
14	—	40.49	**6′′**		61.51	**6′′′′′**	3.46 (m), 3.72 (m)	61.19
15	1.25, 1.56 (m)	35.72						
16	4.32 (br s)	72.48	**GlcII**			**Rha I**		
17	—	48.63	**1′′′**		**106.54** a	**1′′′′′′**	5.40 (s)	**99.41** a
18	2.7 (br s)	41.16	**2′′′**	4.17 (d, 7.5)	73.62	**2′′′′′′**	3.31 (m)	72.48
19	0.97, 2.22 (m)	46.48	**3′′′**	3.53 (m)	78.77	**3′′′′′′**	3.32 (m)	72.59
20	—	30.18	**4′′′**	3.48 (m)	70.46	**4′′′′′′**	3.80 (m)	**80.57** a
21	1.08, 1.89 (m)	35.00	**5′′′**	3.54 (m)	76.77	**5′′′′′′**	3.56 (m)	70.07
22	1.23, 1.38 (m)	32.31	**6′′′**	3.25 (m)	61.06	**6′′′′′′**	1.05 (d, 6.01)	17.86
23	—	180.73		3.43 (m), 3.63 (m)				
24	1.14 (s)	13.81				**RhaII**		
25	1.18 (s)	16.39				**1′′′′′′′**	5.26 (s)	**99.10** a
26	0.66 (s)	16.49				**2′′′′′′′**	3.35 (m)	72.40
27	1.28 (s)	26.45				**3′′′′′′′**	3.33 (m)	72.33
28	—	175.34				**4′′′′′′′**	3.46 (m)	73.20
29	0.83 (s)	32.86				**5′′′′′′′**	3.52 (m)	70.10
30	0.90 (s)	24.24				**6′′′′′′**	1.10 (d, 6.42)	18.10

a The site of the link.

Additionally, the ^1^H-NMR spectrum of 1 revealed the presence of seven anomeric protons at *δ*_H_ 4.17, 4.19, 4.21, 4.25, 5.19, 5.26 and 5.40, which correlated with *δ*_C_ 106.54, 103.20, 103.18, 105.76, 93.27, 99.10 and 99.41, respectively, from the HSQC spectrum and indicated the presence of seven *O*-linked sugar moieties. Taken together with the MS data, these suggested that 1 was a triterpenoid saponin with seven sugars. Acid hydrolysis of 1 gave four sugar types deduced to be arabinose, rhamnose, xylose, and glucose using co-paper chromatography in comparison with authentic sugars. A full set of ^1^H and ^13^C NMR assignments for each sugar residue was derived from a combination of COSY, HSQC and HMBC data. The sugars present were identified as glucose I at *δ*_H_ 4.19 (1H, d, *J* = 7.21 Hz, H-1 of Glc I), glucose II at *δ*_H_ 4.17 (1H, d, *J* = 7.5 Hz, H-1 of Glc II), glucose III at *δ*_H_ 4.21 (1H, d, *J* = 7.34 Hz, H-1 of Glc III), xylose at *δ*_H_ 4.25 (1H, d, *J* = 7.00 Hz, H-1 of Xyl), arabinose at *δ*_H_ 5.19 (1H, br s, H-1 of Ara), rhamnose I at *δ*_H_ 5.40 (1H, br s, H-1 of Rha I) and rhamnose II at *δ*_H_ 5.26 (1H, br s, H-1 of Rha II). The β anomeric configurations of the d-glucose and d-xylose units were determined from their coupling constants (7.0–7.5 Hz). The α-anomeric configurations of l-arabinose and l-rhamnose were determined based on their broad singlet signals of anomeric protons at *δ*_H_ 5.19, 5.26, and 5.40 in the ^1^H-NMR spectra.

**Fig. 2:**
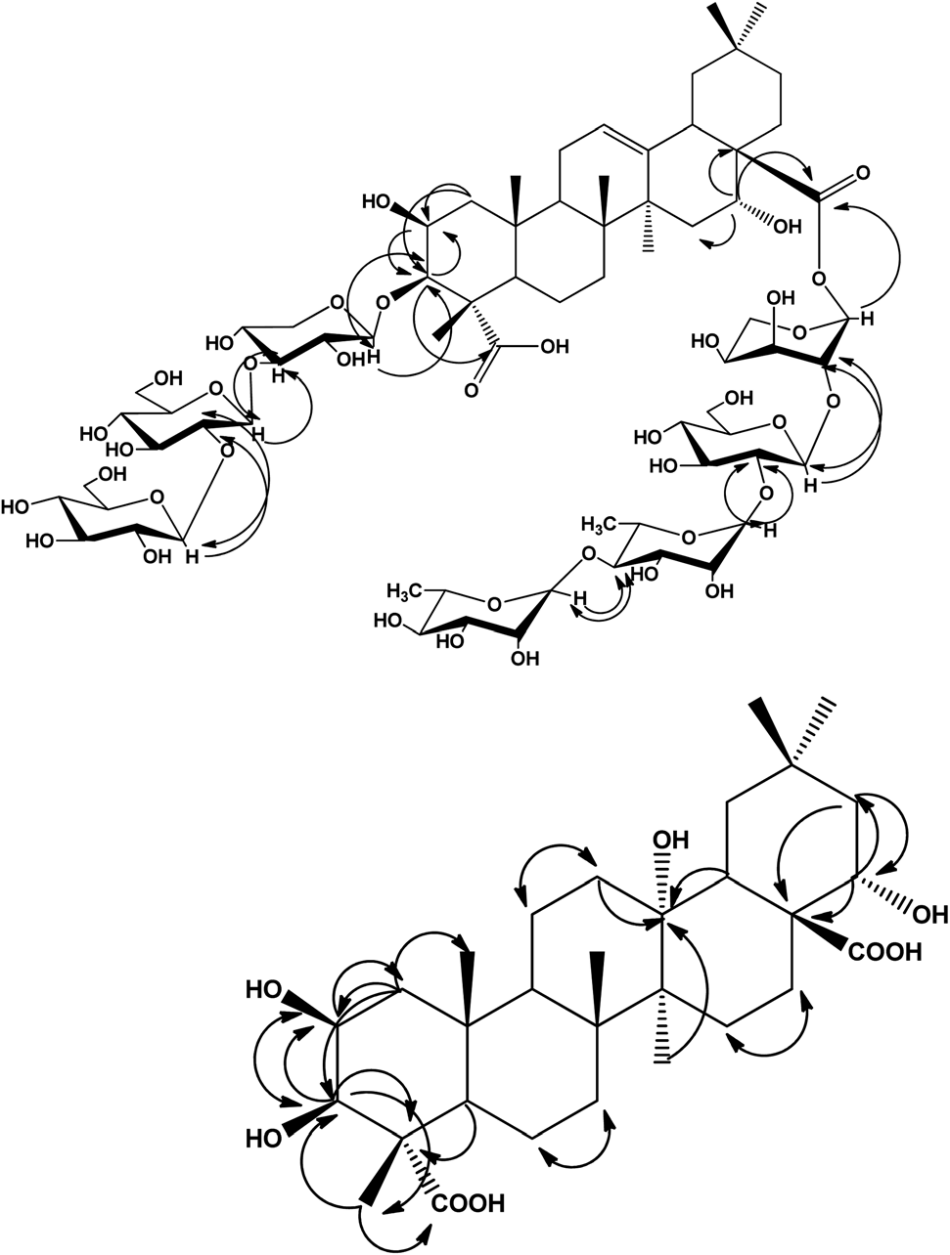
Significant HMBC and NOESY correlations of compounds 1 and 2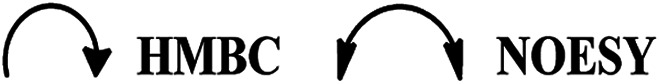.

The HMBC correlations between the aglycone and the sugar resonances indicated that compound 1 was a bisdesmoside. The long range correlations were observed at C-3 between H-3 of aglycone at *δ*_H_ 3.81 and C-1 of xylose at *δ*_C_ 105.76, and from H-1 of Xyl at *δ*_H_ 4.25 to C-3 at *δ*_C_ 80.40. In addition, the downfield shifted resonances of C-3 at *δ*_C_ 84.20 and H-3 at *δ*_H_ 4.05 of xylose indicated further substitution at this position, which was confirmed by the HMBC correlations of H-1 at *δ*_H_ 4.19 of glucose I to C-3 of xylose at *δ*_C_ 84.20, and H-3 of xylose at *δ*_H_ 4.05 to C-1 of glucose I at *δ*_C_ 103.20. Furthermore, the downfield shifted resonances of C-2 (*δ*_C_ 81.60) and H-2 (*δ*_H_ 3.78) of glucose I indicated further substitution at this position, as confirmed by the HMBC correlations (Fig. 2) from H-2 of glucose I at *δ*_H_ 3.78 to C-1 of glucose II at *δ*_C_ 106.54, and H-1 of glucose II at *δ*_H_ 4.17 to C-2 of glucose I at *δ*_C_ 81.60. Thus, the β-d-glucopyranosyl-(1 → 2)-β-d-glucopyranosyl-(1 → 4)-β-d-xylopyranosyl moiety was *O*-linked at C-3. The remaining four sugar moieties were present as a tetrasaccharide *O*-linked at C-28. The primary sugar was arabinose, whereas the HMBC spectrum showed a correlation between its anomeric proton at *δ*_H_ 5.19 and C-28 at *δ*_C_ 175.34. The other sugar sequence was also confirmed from HMBC correlations, whereas a significant correlation was observed between the anomeric proton at *δ*_H_ 4.21 of glucose III and C-2 of arabinose at *δ*_C_ 75.70. Another HMBC correlation was exhibited between H-1 at *δ*_H_ 5.40 of rhamnose I with C-2 of glucose III at *δ*_C_ 81.95, in addition to the correlation of H-1 of rhamnose II at *δ*_H_ 5.26 with C-4 at *δ*_C_ 80.57 of the rhamnose I moiety. From the previously mentioned data and comparison with the literature,^9,21,23–29^ compound 1 was determined to be 3-*O*-[β-d-glucopyranosyl-(1 → 2)-β-d-glucopyranosyl-(1 → 4)-β-d-xylopyranosyl]-2β,3β,16α-trihydroxyolean-12-en-23,28-dioic acid-28-*O*-α-l-rhamnopyranosyl-(1 → 4)-α-l-rhamnopyranosyl-(1 → 2)-β-d-glucopyranosyl-(1 → 2)-α-l-arabinopyranoside, which is a new compound.

Compound 2 was isolated as a white amorphous powder. Its molecular formula was established as C_30_H_48_O_8_ from HR-ESI-MS. The IR spectrum of 2 showed absorption bands at 3452 cm^−1^ for the hydroxyl groups, 1733 cm^−1^ for the carbonyl groups and 1389 cm^−1^ for the gem-dimethyl. The ^1^H-NMR spectrum of compound 2 showed six singlet signals for the six methyl groups at *δ*_H_ 0.69, 0.86, 1.20, 0.83, 1.11, 1.14, which were attributed to H_3_-30, H_3_-29, H_3_-27, H_3_-26, H_3_-25 and H_3_-24, respectively. The ^1^H-NMR also showed three broad singlet signals at *δ*_H_ 3.91, 3.73 and 3.95 for the three oxygenated methine protons, which were confirmed from the ^13^C-NMR signals at *δ*_C_ 70.11, 74.65 and 69.81. These signals were assigned to the oxymethines C-2, C-3 and C-22, respectively.

Additionally, the ^13^C-NMR spectrum displayed a signal at *δ*_C_ 69.12, which was attributed to an oxyquaternary carbon (C-13), in addition to two carbonyl carbon signals at *δ*_C_ 179.16 and 177.46 that were assigned to C-23 and C-28, respectively. The ^13^C-NMR spectrum of compound 2 did not display any olefinic carbons, indicating the absence of unsaturation at C-12. The aforementioned spectral data, together with physicochemical properties, revealed the oleanane triterpene nature of compound 2. The localization of the hydroxyl groups was confirmed from the HMBC spectrum that showed significant correlations between H-1 at *δ*_H_ 1.17, 2.05 with C-2 at *δ*_C_ 70.11 and C-3 at *δ*_C_ 74.65. In addition, H-3 at *δ*_H_ 3.73 was correlated to C-2 at *δ*_C_ 70.11, C-4 at *δ*_C_ 52.30, and C-23 at *δ*_C_ 179.16 and C-24 at *δ*_C_ 12.46. H-24 at *δ*_H_ 1.14 showed a long range correlation with C-3 at *δ*_C_ 74.65, C-4 at *δ*_C_ 52.30, C-5 at *δ*_C_ 51.22 and C-23 at *δ*_C_ 179.16. This established the attachment of the two secondary hydroxyl groups at C-2 and C-3 of ring A, and confirmed the presence of a carboxylic group at C-23. Likewise, H-22 at *δ*_H_ 3.95 showed a correlation with C-21 at *δ*_C_ 44.35, while H-21 at *δ*_H_ 1.18, 2.32 showed correlations with C-17 at *δ*_C_ 48.63, C-20 at *δ*_C_ 33.12 and C-22 at *δ*_C_ 69.81, revealing the presence of another hydroxyl group attached at C-22. The fourth hydroxyl group was deduced to directly attach to C-13 from the HMBC correlations between H-12 at *δ*_H_ (1.36, 2.15) and C-13 at *δ*_C_ 69.12. Other correlations included H-27 at *δ*_H_ 1.20 and C-13 at *δ*_C_ 69.12, and H-18 at *δ*_H_ 1.72 and C-13 at *δ*_C_ 69.12, as shown in Fig. 2. The COSY spectrum showed vicinal couplings between H-2 at *δ*_H_ 3.91 with methine H-3 at *δ*_H_ 3.73 and methylene H-1 protons at *δ*_C_ (1.17 and 2.05). Additionally, significant correlations exhibited between H-18 (*δ*_H_ 1.72)/H-19 (*δ*_H_ 1.22, 1.39), and H-11 (1.16, 1.51)/H-12 (1.36, 2.15) were also observed. The direct single bond ^1^H/^13^C connectivity of each protonated carbon was deduced with the help of the HMQC data (Table 2). From the above spectral observations and comparison with the literature of related compounds,^30–32^ compound 2 was identified as 2β,3β,13α,22α-tetrahydroxy oleanane-23,28-dioic acid, which is a new natural product that was first isolated and identified here.

**Table 2:** ^1^H and ^13^C spectral data of compound 2 (600 MHz and 150 MHz, respectively, DMSO-*d*_6_)

No.	*δ*_H_ (m, *J* in Hz)	*δ*_C_
1	1.17, 2.05 (m)	44.62
2	3.91 (br s)	70.11
3	3.73 (br s)	74.65
4	—	52.30
5	1.41 (t)	51.22
6	1.02, 1.47 (m)	20.68
7	1.19, 1.76 (m)	35.38
8	—	41.97
9	1.48 (br s)	50.80
10	—	39.20
11	1.16, 1.51 (m)	22.14
12	1.36, 2.15 (m)	33.97
13	—	69.12
14	—	52.75
15	1.65, 1.78 (m)	26.54
16	1.86, 2.60 (m)	24.93
17	—	48.63
18	1.72 (m)	41.38
19	1.22, 1.39 (m)	36.05
20	—	33.12
21	1.18, 2.32 (m)	44.35
22	3.95 (br s)	69.81
23	—	179.16
24	1.14 (s)	12.46
25	1.11 (s)	17.49
26	0.83 (s)	18.15
27	1.20 (s)	24.99
28	—	177.46
29	0.86 (s)	32.44
30	0.69 (s)	24.43

### 2.2. Identification of known compounds

The other isolated compounds were identified based on various spectroscopic methods, including 1D and 2D NMR experiments (^1^H-NMR, ^13^C-NMR, DEPT, HSQC, and HMBC), as 2β,3β,16α-trihydroxy olean-23,28-dioic (zanhic acid) (3),^22^*p*-hydoxybenzaldehyde (6)^33^ and spinasterol-3-*O*-β-d-glucopyranoside (5),^34,35^ which are reported for the first time in the plant. In addition, 2β,3β-dihydroxyolean-12-ene-23,28-dioic (medicagenic acid) (4),^4^*p*-hydroxymethylcinnamate (7),^36^ and desoxyerytholaccacin (8)^10^ were identified.

### 2.3. Biological investigation of the isolated compounds

The saponin fraction and the isolated compounds 1–8 were tested for their tumor cell growth inhibitory activity against five human cancer cell lines, PC-3, A-549, HePG-2, MCF-7 and HCT-116. The saponin fraction exhibited potent cytotoxic activity against all tested cell lines, whereas the maximum activity was observed against prostate cancer cell line (PC-3) and lung carcinoma cell line (A-549) with IC_50_ values of 1.13 and 1.98 μg mL^−1^, respectively. Futhermore, it showed good activity against the hepatocellular carcinoma (HePG2) and breast adenocarcinoma (MCF-7) cell lines with IC_50_ values of 3.01 and 4.23 μg mL^−1^, respectively. The biological results indicated that compound 1 was the most active among the isolated compounds. It showed potent activity against A-549 and PC-3 with IC_50_ values of 2.41 μg mL^−1^ and 3.45 μg mL^−1^, respectively. Compound 1 also showed moderate activity against the HePG2 and MCF-7 cell lines with IC_50_ values of 5.98 and 6.43 μg mL^−1^, respectively.

Compound 2 showed the maximum activity against PC-3 with an IC_50_ value of 2.01 μg mL^−1^, followed by A-549 (IC_50_ 5.01 μg mL^−1^), in comparison to the cytotoxic control drug adriamycine (doxorubicin), which exhibited IC_50_ 1.37, 1.63, 2.26, and 2.65 against A-549, PC-3, MCF-7, and HePG2, respectively, as shown in Table 3.

**Table 3:** Results of the cytotoxic activity of the saponin fraction and some isolated compounds from *Gladiolus segetum* Ker-Gawl

Cpd	IC_50_ (μg mL^−1^)				
	PC-3	HePG-2	A-549	MCF-7	HCT-116
Saponin fraction	1.13 ± 0.31	3.01 ± 0.36	1.98 ± 0.33	4.23 ± 0.42	9.77 ± 0.77
1	3.45 ± 0.26	5.98 ± 0.21	2.41 ± 0.34	6.43 ± 0.23	>50
2	2.01 ± 0.23	6.82 ± 0.35	5.01 ± 0.26	>50	>50
3	10.8 ± 0.72	15.7 ± 0.78	11.5 ± 0.25	>50	>50
4	10.7 ± 0.68	14.4 ± 0.72	12.7 ± 0.58	>50	>50
5	>50	>50	>50	>50	>50
6	>50	>50	>50	>50	>50
7	>50	>50	>50	>50	>50
8	>50	>50	>50	>50	>50
Doxorubicin	1.63 ± 0.22	2.65 ± 0.32	1.37 ± 0.21	2.26 ± 0.28	3.86 ± 0.31

From these aforementioned results, it is obvious that the cytotoxic potency of the triterpenoid saponin 1 is higher than that of the triterpene aglycone 2, which was in harmony with earlier studies.^37–39^ This clarified the important structural feature of the presence of an oligosaccharide ester at C-3 and a free hydroxyl group at C-16, which were considered to be essential for the cytotoxic activity. Many published studies from literature revealed the correlation of the cytotoxic activity of saponins with the presence of the C-2 glycoside linkage of the first sugar. This could induce the electron deficiency near C-3 of the aglycone more than at the other sites of the first sugar, together with the several types of functional groups. Actually, there are a number of saponins that have occurred in natural sources with a (1 → 2) glycoside linkage between the first and second sugars, rationalizing the wide abundance of this type of saponin.^40,41^ Concerning triterpene aglycone, compound 2 showed cytotoxic activity higher than 5, which may attributed to the presence of a free carboxyl group at C-28.^39,41^

### 2.4. Molecular modeling

Molecular modeling studies play an important role in order to construct molecular models that help in better understanding the binding mode of target compounds at the molecular level. Therefore, compounds 2, 3, 4, 5, and 8 were selected to be docked in the active site of human epidermal receptor 2 (HER-2) at the catalytic ligand binding site (PDBID: 5jeb), which is overexpressed in several epithelial-derived tumors including breast, ovarian, lung, renal, colorectal and brain. HER-2 is a family of receptors that plays a vital role in the pathogenesis of several human cancers. They regulate cell growth, survival, and differentiation *via* multiple signal transduction pathways and participate in cellular proliferation and differentiation. Therefore, HER-2 receptors overexpressed in tumor cells represent an active target for various cancer therapeutics.^18,20,42^

**Fig. 3:**
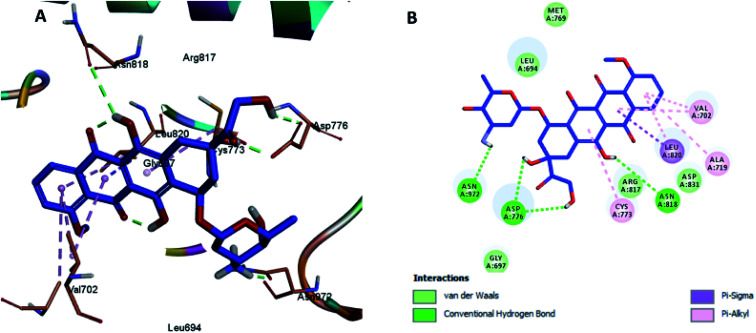
Docking and binding pattern of DOX in the active site of the HER-2 receptor (PDB entry: 5jeb) (blue). (A) 3D structure of DOX, (B) 2D structure of DOX.

**Fig. 4:**
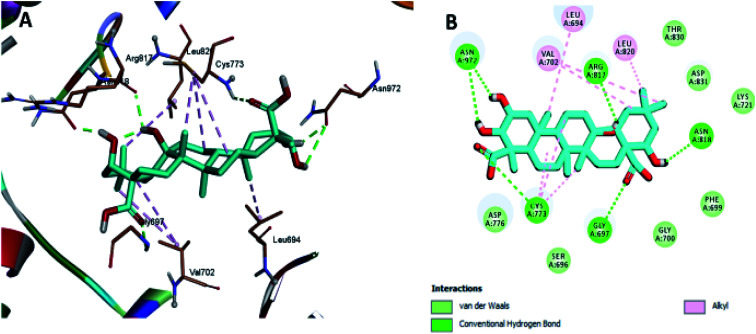
Docking and binding pattern of compound 2 in the active site of the HER-2 receptor (PDB entry: 5jeb) (cyan). (A) 3D structure of compound 2, (B) 2D structure of compound 2.

**Fig. 5:**
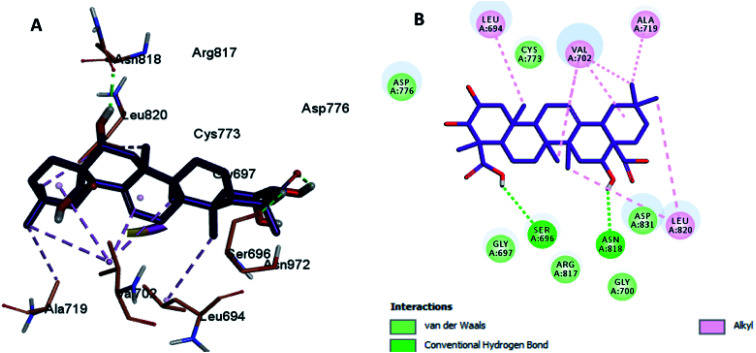
Docking and binding pattern of compound 3 in the active site of the HER-2 receptor (PDB entry: 5jeb) (violet). (A) 3D structure of compound 3, (B) 2D structure of compound 3.

**Fig. 6:**
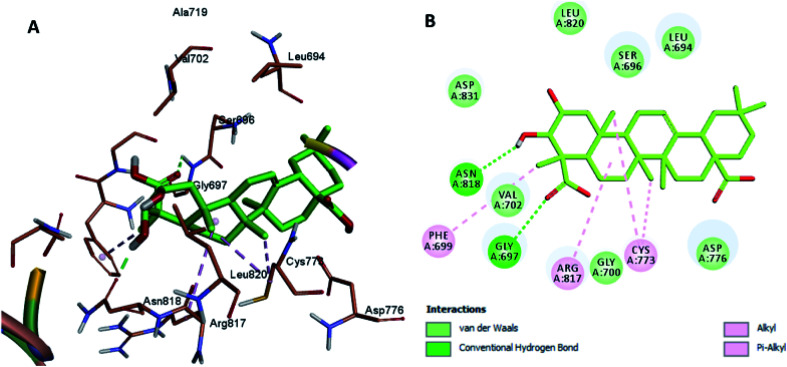
Docking and binding pattern of compound 4 in the active site of the HER-2 receptor (PDB entry: 5jeb) (green). (A) 3D structure of compound 4, (B) 2D structure of compound 4.

**Fig. 7:**
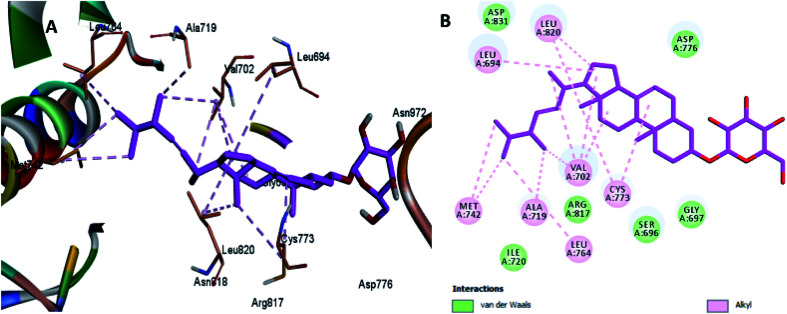
Docking and binding pattern of compound 5 in the active site of the HER-2 receptor (PDB entry: 5jeb) (pink). (A) 3D structure of compound 5, (B) 2D structure of compound 5.

**Fig. 8:**
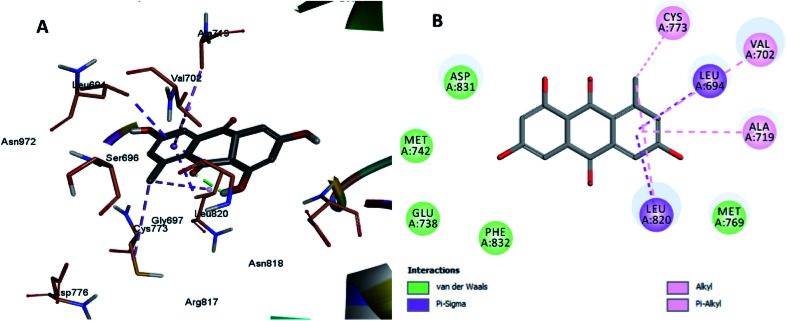
Docking and binding pattern of compound 8 in the active site of the HER-2 receptor (PDB entry: 5jeb) (grey). (A) 3D structure of compound 8, (B) 2D structure of compound 8.

Herein, we report the binding pose of DOX and compounds 2, 3, 4, 5, and 8 with HER-2 protein, as shown in the form of ligplots depicted in Fig. 3–8(A) and (B) using the Discovery Studio software package, to elucidate the binding mode of these compounds with HER-2 protein and investigate their similarity to DOX. The molecular docking study of DOX against the HER-2 protein receptor (Organism: human), revealed a common binding orientation of DOX in the catalytic binding pocket of the HER-2 protein receptor (PDBID: 5jeb). As a first step, for the validation of the docking parameters, the co-crystal ligand (6JS) was re-docked at the catalytic site of the protein. The RMSD between the co-crystal and re-docked poses was found to be 0.331(<2 Å), which confirmed the validity of the docking parameters.^43^

The docking results revealed that the binding interaction of DOX with the backbone of the HER-2 protein receptor (Fig. 3) show four hydrogen bond interactions with Asp 776, Asn 818 and Asn 972, in addition to the many hydrophobic interactions with the different amino acid residues within the pocket (*i.e.*, Val 702, Ala 719, Cys 773 and Leu 820). The docking results of compound 2 indicated that it bound nicely to the catalytic binding pocket of the HER-2 protein receptor (PDBID: 5jeb) (Fig. 2) *via* incorporation of five hydrogen bonds with Gly 697, Cys 773, Arg 817, Asn 818 and Asn 972, in addition to the many hydrophobic interactions with the different amino acid residues within the pocket (*i.e.*, Leu 694, Val 702, Cys 773 and Leu 820) (Fig. 4).

Moreover, the docking results of compounds 3 and 4 (Fig. 5 and 6) showed that they incorporated two hydrogen bonds only with Ser 696 and Asn 818 for compound 3, and with Gly 697 and Asn 818 for compound 4, respectively, in addition to the many hydrophobic interactions with different amino acid residues, such as Phe 699, Cys 773, Arg 817, Leu 694, Val 702, Ala 719, and Leu 820.

The least active compounds 5 and 8 (Fig. 7 and 8) show only hydrophobic interactions with different amino acid residues, such as Phe 699, Cys 773, Arg 817, Leu 694, Val 702, Ala 719, Leu 694, Met 742, Leu 764 and Leu 820.

In conclusion, these results indicate that the anticancer activities of these compounds are in agreement with their biological results. Our results strongly suggest that the possible mechanism of action of these compounds, in particular that of compound 2, may be through the inhibition of the HER-2 enzyme.

## 3. Experimental

### 3.1. General

The ESI-MS of compound 1 were measured on ultrahigh resolution mass spectra acquired with a solariX 7.0 T equipped with an ESI/MALDI dual ion source (Bruker Daltonics, Bremen, Germany) in the positive ion mode. Compound 2 was measured on a Shimadzu LCMS-IT-TOF (Japan). The FTIR spectra were measured on a Shimadzu Infra-red-470 spectrophotometer (Japan). NMR analyses (^1^H-NMR, COSY, NOESY, ^13^C-NMR, DEPT-^13^C-NMR HSQC, HMBC) were measured on a Bruker Avance III HD 600 MHz spectrometer (Japan) and a Varian mercury 400 MHz Spectrometer (Japan) using TMS as the internal standard. Column chromatography was carried on Silica Gel 60 N, 40–50 μm (pH: 7.0 ± 0.5), Sephadex LH-20 (20–100 μm) and Wakogel 50C18 (38–63 μm) (Wako Chemical Co., Ltd., Japan). HPLC was performed using a Mightysil RP-18 column (250–20, 5 μm) on a LC-8A preparative liquid chromatography with a SPD-10A UV detector (Shimadzu, Japan) and SCL-10A Analytical HPLC using ODS-120T column and Waters 2487 dual UV detector (Shimadzu, Japan). TLC analyses were performed on precoated Kieselgel 60F_254_ plates (Merck, KGaA 64271, Germany) and TLC precoated Kieselgel 60 RP-18 F_254_ s plates (Merck, KGaA 64271, Germany). The plates were examined under UV light (at 365 and 254 nm) and visualized by spraying with an anisaldehyde reagent, followed by heating at 110–140 °C for 1–2 min, and allowed to dry at room temperature. The following solvent systems were used for TLC:

**Table:** 

I – *n*-Hexane–EtOAc (7 : 3 v/v)	IV – CH_2_Cl_2_–MeOH (8.5 : 1.5 v/v)
II – CH_2_Cl_2_–MeOH (9.5 : 0.5 v/v)	V – CH_2_Cl_2_–MeOH-1% aqu. TFA (7 : 4 : 1 v/v)
III – CH_2_Cl_2_–MeOH (9 : 1 v/v)	VI – Toluene–(CH_3_)_2_C=O–HCHO (3 : 6 : 1 v/v)

### 3.2. Plant material

The corms of *Gladiolus segetum* Ker-Gawl were collected from the fields of Giza governorate (2012), and kindly identified and authenticated by Prof. Dr Naeam Keltawy, Professor of Horticultures, Department of Horticulture, Faculty of Agriculture, Assiut University, Assiut, Egypt. A voucher specimen (Gs-002) was kept in the Department of Pharmacognosy, Faculty of Pharmacy, Al-Azhar University, Assiut, Egypt.

### 3.3. Acid hydrolysis and authentic sugar materials

Compound 1 (4 mg) was refluxed in 10 mL of 1 N HCl for 4 h. Aglycone was extracted with methylene chloride. The water soluble fraction was neutralized with Na_2_CO_3_, and sugars in the aqueous solution were identified by co-paper chromatography (PC) with authentic materials using *n*-BuOH–AcOH–H_2_O (4 : 1 : 5, upper layer) using an aniline phthalate spray as the detection reagent, and their data (*R*_f_) revealed the presence of Glc (*R*_f_ 0.13), Arb (*R*_f_ 0.16), Xylose (*R*_f_ 0.22) and Rha (*R*_f_ 0.28) units.

d-Glucose, d-xylose, l-rhamnose and l-arabinose were obtained from Wako Chemical Co., Ltd., Japan.

### 3.4. Cell lines, culture media and chemicals

Human prostate cancer (PC-3), human lung carcinoma (A-549), human hepatocellular carcinoma (HePG-2), human breast adenocarcinoma (MCF-7) and human colon cell line (HC-T116) were purchased from the American Tissue Culture Collection. Dulbecco's Modified Eagle's Medium (DMEM), Roswell Park Memorial Institute medium (RPMI 1640), and other chemicals were obtained from Sigma-Aldrich Co. (Baden-Württemberg, Germany).

### 3.5. Extraction and isolation

The air-dried powdered corms (800 g) of *Gladiolus segetum* Ker-Gawl were extracted by maceration and percolation with ethanol until complete exhaustion. The ethanolic extract (78.0 g) was subjected to successive solvent fractionation over VLC with chloroform (15.0 g), *n*-butanol saturated with water (40.0 g), and finally washed with methanol (20.0 g) (4 L in each solvent).

The butanolic extract was dissolved in MeOH (400.0 mL), and added portionwise into cold diethyl ether (2 L). The precipitate was removed by filtration on a Buchner funnel, washed with cold diethyl ether, and dried under reduced pressure to give the saponin fraction (32 g).

Part of the *n*-butanol (saponin) fraction (500.0 mg) was chromatographed over RP-18, eluted by gradient with MeOH–H_2_O. Fractions 15–20 were subjected to further purification by HPLC and eluted with CH_3_CN–H_2_O (34%), and UV detection at 205 nm resulted in the separation of compound 1 (8.0 mg) with a running time of 33 min.

A part of the chloroform-soluble fraction (10 g) was chromatographed over a silica gel column, eluted with CH_2_Cl_2_ and followed by a CH_2_Cl_2_–MeOH gradient elution, affording the GC1-GC4 sub-fractions. Sub-fraction GC3 (150.0 mg) was chromatographed over RP-18, and eluted with MeOH–H_2_O (70 : 30). Fractions 33–42 were subjected to further purification by HPLC and eluted with CH_3_CN–H_2_O (59%), and UV detection at 205 nm resulted in the separation of compounds 2 (12.0 mg) with a running time of 16 min. and 3 (10.0 mg; rt. 17 min). Fractions 22–27 afforded compound 4 (15.0 mg). Likewise, sub-fraction GC-4 (100.0 mg) was chromatographed on a silica gel column eluted with CH_2_Cl_2_–MeOH, and afforded compound 5 (50.0 mg).

Sub-fraction GC2 (200.0 mg) was chromatographed over sephadex LH-20 using CH_2_Cl_2_–MeOH (1 : 1). Fractions 8–12 were concentrated under reduced pressure afforded compound 6 (10.0 mg), while fractions 16–20 were collected and concentrated under reduced pressure, affording compound 7 (30.0 mg). Fractions 25–32 were rechromatographed over silica gel column chromatography, and eluted isocratically with CH_2_Cl_2_–MeOH (9.5 : 0.5), resulting in the separation of 8 (40.0 mg).

#### 3.5.1. 3-*O*-[β-d-Glucopyranosyl-(1 → 2)-β-d-glucopyranosyl-(1 → 4)-β-d-xylopyranosyl]-2β,3β,16α-trihydroxyolean-12-en-23,28-dioic acid-28-*O*-α-l-rhamnopyranosyl-(1 → 4)-α-L rhamn opyranosyl-(1 → 2)-β-d-glucopyranosyl-(1 → 2)-α-l-arabinopyranoside (1)

Compound 1 was obtained as a white amorphous powder with *R*_f_ = 0.30 (system, V) and IR (KBr) cm^−1^: 3410, 2929, 1737, 1637, 1039. HRESI-MS showed a molecular ion peak at *m*/*z*: 1583.6663 [M + Na]^+^ (Calcd for C_70_H_112_O_38_Na: 1583.6729). MS/MS (MS^2^) of [M + Na]^+^ gave fragments at *m*/*z*, 997.45753 = [(M + Na)-132-146-146-162]^+^ attributed to the loss of a tetraglycosidic chain comprising one pentose, two deoxyhexoses and one hexose. The ^1^H-NMR (600 MHz, DMSO-*d*_6_) and ^13^C-NMR (150 MHz, DMSO-*d*_6_) spectral data are compiled in Table 1.

#### 3.5.2. 2β,3β,13α,22α-Tetrahydroxy-oleanane-23,28-dioic acid (2)

Compound 2 was obtained as a white amorphous powder with *R*_f_ = 0.34 (system, III). The positive ion mode HR-ESI-MS spectrum showed fragments [M + Na–H_2_O]^+^ at *m*/*z* = 541.3121 and [M + 1-2COOH–2OH]^+^ at *m*/*z* = 413.2653, while the negative ion mode showed the fragment [M − Na–H_2_O]^−^ at *m*/*z* = 517.3124. The ^1^H-NMR spectral data (600 MHz, DMSO-*d*_6_) and ^13^C-NMR spectral data (DMSO-*d*_6_, 150 MHz) are listed in Table 2.

#### 3.5.3. 2β,3β,16α-Trihydroxyolean-12-ene-23,28-dioic (zanhic) acid (3)

Compound 3 was obtained as a white amorphous powder with *R*_f_ = 0.36 (system, III). The GC-MS spectrum showed the molecular ion peak at *m*/*z* [M]^+^ = 518, coincident with the molecular formula C_30_H_46_O_7_. ^1^H-NMR spectral data (300 MHz, DMSO-*d*_6_) *δ*_H_: 0.67, 0.82, 0.89, 1.16, 1.17 and 1.29 (each 3H, s, CH_3_ of C-26, C-29, C-30, C-24, C-25 and C-27), 3.73 (1H, m, H-3), 3.88 (1H, m, H-2), 4.30 (1H, br s, H-16), 5.20 (1H, br s, H-12). ^13^C-NMR spectral data (DMSO-*d*_6_, 150 MHz) *δ*_C_: 44.04 (C-1), 69.99 (C-2), 74.73 (C-3), 52.10 (C-4), 51.10 (C-5), 20.45 (C-6), 34.58 (C-7), 39.92 (C-8), 47.33 (C-9), 35.87 (C-10), 22.97 (C-11), 121.18 (C-12), 144.03 (C-13), 40.04 (C-14), 35.86 (C-15), 72.96 (C-16), 46.67 (C-17), 41.17 (C-18), 46.44 (C-19), 30.26 (C-20), 35.18 (C-21), 32.34 (C-22), 179.50 (C-23), 12.67 (C-24), 16.28 (C-25), 16.77 (C-26), 26.52 (C-27), 178.49 (C-28), 32.93 (C-29) and 24.28 (C-30).^22^

#### 3.5.4. 2β,3β-Dihydroxy-olean-12-ene-23,28-dioic (medicagenic) acid (4)

Compound 4 was obtained as a white amorphous powder with *R*_f_ = 0.39 (system, III). The GC-MS spectrum showed the molecular ion peak [M]^+^ at *m*/*z* = 502 coincident with the molecular formula C_30_H_46_O_6_. Selected ^1^H-NMR spectral data (600 MHz, DMSO-*d*_6_) *δ*_H_: 0.69, 0.86, 1.01, 1.16, 1.86 and 1.91 (each 3H, s, CH_3_ of C-26, C-29, C-30, C-24, C-25 and C-27), 3.73 (1H, m, H-3), 3.88 (1H, m, H-2), 5.16 (1H, br s, H-12). ^13^C-NMR spectral data (DMSO-*d*_6_, 150 MHz) *δ*_C_: 43.95 (C-1), 69.95 (C-2), 74.47 (C-3), 52.15 (C-4), 50.92 (C-5), 20.41 (C-6), 33.81 (C-7), 40.79 (C-8), 47.55 (C-9), 35.87 (C-10), 22.59 (C-11), 121.51 (C-12), 143.74 (C-13), 40.13 (C-14), 27.09 (C-15), 23.35 (C-16), 45.42 (C-17), 41.41(C-18), 45.69 (C-19), 30.37 (C-20), 35.82 (C-21), 32.07(C-22), 179.04 (C-23), 12.52 (C-24), 16.19 (C-25), 16.69 (C-26), 25.61 (C-27), 178.57 (C-28), 32.81 (C-29) and 22.95 (C-30).^4^

#### 3.5.5. Spinasterol-3-*O*-β-d-glucopyranoside (5)

Compound 5 was obtained as a white amorphous powder, which exhibited *R*_f_ = 0.36 (system, IV). The GC-MS spectrum showed the molecular ion peak at *m*/*z* 574 [M]^+^ corresponding to the molecular formula C_35_H_58_O_6_, 411 [M-glucose] and other peaks at 361, 301, 278, 258, 238, 218, 185, 167, 149, 147, 125, 106 and 77. The selected ^1^H-NMR spectra (400 MHz, DMSO-*d*_6_) showed the following signals: *δ*_H_ 0.50 (3H, br s, H-18); 0.73 (3H, br s, H-19); 0.76 (3H, br s, H-27); 0.81 (3H, br s, H-29); 0.99 (3H, br s, H-26); 1.04 (3H, br s, H-21); 4.21 (1H, m, H-3); 4.89 (1H, d, *J* = 7.2 Hz, H-1′); 5.11 (1H, m, H-23); 5.32 (1H, br s H-7); 5.44 (1H, m, H-22). The ^13^C-NMR (100 MHz, DMSO-*d*_6_) spectral data showed signals at *δ*_C_: 36.49 (C-1), 30.86 (C-2), 89.85 (C-3), 34.91 (C-4), 40.11 (C-5), 29.07 (C-6), 117.16 (C-7), 139.05 (C-8), 48,62 (C-9), 33.91 (C-10), 21.12 (C-11), 39.70 (C-12), 42.63 (C-13), 54.23 (C-14), 22.44 (C-15), 28.3 (C-16), 55.18 (C-17), 12.14 (C-18), 13.95 (C-19), 39.89 (C-20), 18.85 (C-21), 137.96 (C-22), 128.88 (C-23), 50.64 (C-24), 31.32 (C-25), 20.99 (C-26), 17.90 (C-27), 24.82 (C-28) and 12.63 (C-29).^34,35^

#### 3.5.6. *p*-Hydoxybenzaldehyde (6)

Compound 6 was obtained as a white amorphous powder, which showed *R*_f_ = 0.43 (system, II). The GC-MS spectrum showed the molecular ion peak [M]^+^ at *m*/*z* = 138, which was consistent with the molecular formula C_6_H_7_O_2_. ^1^H-NMR spectral data (CD_3_OD, 600 MHz): *δ*_H_ 6.92 (each, 1H, d, *J* = 8.6 Hz, H-4, 6), 7.77 (each, 1H, d, *J* = 8.6 Hz, H-3, 7) and 9.51 (1H, s, H-1). ^13^C-NMR spectral data (CD_3_OD, 150 MHz): *δ*_C_ 193.06 (C-1), 130.26 (C-2), 133.49 (C-3), 116.92 (C-4), 165.2 (C-5) 116.92 (C-6) and 133.49 (C-7).^33^

#### 3.5.7. *p*-Hydroxymethylcinnamate (7)

Compound 7 was obtained as a white amorphous powder with *R*_f_ = 0.38 (system, II). The GC-MS spectrum showed the molecular ion peak [M]^+^ at *m*/*z* = 178 calculated to the molecular formula C_10_H_10_O_3_. ^1^H-NMR spectral data (CD_3_OD, 600 MHz): *δ*_H_ 3.3 (3H, s, H_3_–OCH_3_), 6.33 (1H, d, *J* = 15.9 Hz, H-2), 6.81 (each, 1H, d, *J* = 8.6 Hz, H-6&8), 7.45 (each, 1H, d, *J* = 8.6 Hz, H-5 and 9) and 7.60 (1H, d, *J* = 15.9 Hz, H-3). ^13^C-NMR spectral data (CD_3_OD, 150 MHz): *δ*_C_ 169.9 (C-1), 114.9 (C-2), 146.6 (C-3), 127.15 (C-4), 131.15 (C-5) 116.86 (C-6), 161.2 (C-7), 116.86 (C-8), 131.15 (C-9) and 52.08 (OCH_3_).^36^

#### 3.5.8. Desoxyerytholaccacin (8)

Compound 8 was obtained as yellow crystals and showed *R*_f_ = 0.34 (system, II). The GC-MS spectrum showed the molecular ion peak [M]^+^ at *m*/*z* = 272 coincident with the molecular formula C_15_H_12_O_5_. ^1^H-NMR spectral data (CD_3_OD, 600 MHz): *δ*_H_ 2.74 (3H, s, H_3_-8), 6.52 (1H, d, *J* = 2.3 Hz, H-2), 6.94 (1H, d, *J* = 2.4 Hz, H-7), 7.08 (1H, d, *J* = 2.3 Hz, H-4) and 7.46 (1H, d, *J* = 2.4 Hz, H-5). ^13^C-NMR spectral data (CD_3_OD, 150 MHz): *δ*_C_ 165.44 (C-1), 109.34 (C-2), 166.34 (C-3), 108.21 (C-4), 113.32 (C-5) 163.20 (C-6), 125.65 (C-7), 138.54 (C-8), 124.50 (C-9), 146.72 (C-10) 136.11 (C-11), 111.79 (C-12) 189.80 (C-13) and 184.38 (C-14).^10^

### 3.6. Antiproliferative activity on human cell lines

#### 3.6.1. Cell cultures

Cells were suspended in RPMI 1640 medium (for HePG-2- MCF-7 and HCT-116) and DMEM medium (for A-549 and PC-3). Media were supplemented with a 1% antibiotic-antimycotic mixture (10 000 U mL^−1^ potassium penicillin, 10 000 mg mL^−1^ streptomycin sulphate and 25 mg mL^−1^ amphotericin B), 1% l-glutamine and 10% fetal bovine serum, according to the cells.

#### 3.6.2. *In vitro* cytotoxic activity (MTT assay)

Cell viability was assessed by the mitochondrial dependent reduction of yellow 3-(4,5-dimethylthiazol-2-yl)-2,5-diphenyl tetrazolium bromide (MTT) to purple formazan, which reflects the normal function of mitochondria and cell viability.^44^ Cells were batch cultured for 10 days, and then seeded at a concentration of 10 × 10^3^ cells per well in freshly prepared complete growth medium in 96-well microtiter plastic plates at 37 °C for 24 h under 5% CO_2_ using a water jacketed carbon dioxide incubator (Sheldon, TC2323, Cornelius, OR, USA). The media was aspirated and fresh medium (without serum) was added, and the cells were incubated either alone (negative control) or with different concentrations of the tested samples to give a final concentration of (100, 50, 25, 12.5, 6.25, 3.125, 1.56 and 0.78 μg mL^−1^). After 48 h of incubation, the medium was aspirated, 40 μL MTT salt (2.5 μg mL^−1^) were added to each well, and the cultures were incubated for four additional hours at 37 °C under 5% CO_2_. To stop the reaction and dissolve the formed crystals, 200 μL of 10% sodium dodecyl sulphate (SDS) in deionized water was added to each well and incubated overnight at 37 °C. A positive control composed of 100 μg mL^−1^ was used as a known cytotoxic natural agent, which gives 100% lethality under the same conditions.^45,46^ The absorbance was then measured using a microplate multi-well reader (Bio-Rad Laboratories Inc., model 3350, Hercules, California, USA) at 595 nm and a reference wavelength of 620 nm. A statistical significance was tested between samples and the negative control (cells with vehicle) using the independent *t*-test by the SPSS 11 program. DMSO was the vehicle used for the dissolution of the plant extracts, and its final concentration on the cells was less than 0.2%. The percentage of change in viability was calculated according to the formula:[(Reading of extract/Reading of negative control) − 1] × 100.

A probit analysis was carried for the IC_50_ and IC_90_ determination using the SPSS 11 program.

### 3.7. *In silico* drug design study: molecular docking

The docking study of DOX and compounds 2, 3, 4, 5, and 8 against the HER-2 receptors was performed using Discovery Studio 2.5 software (Accelrys Inc., San Diego, CA, USA). The docked compounds were sketched in 3D format using the build panel, and were prepared for docking using the ligprep application. The protein for the docking study was taken from the protein data bank (PDB ID: 5jeb). It was prepared by removing the solvent, adding hydrogens and further minimized in the presence of the bound ligand (6JS) using the protein preparation wizard. Grids for molecular docking were generated with the bound co-crystallized ligand.^43,47,48^ Force fields were applied on the docked compounds to get the minimum lowest energy structure. The obtained poses were studied and the poses showing the best ligand – HER-2 interactions were selected and used for the CDOCKER energy (protein–ligand interaction energies) calculations. Receptor–ligand interactions of the complexes were examined in 2D and 3D styles.

## 4. Conclusion

Our study presented the phytochemical investigation of *Gladiolus segetum* Ker-Gawl corms, which led to the isolation and characterization of two new compounds and six known compounds. This study revealed the various classes of secondary metabolites in the plant. The structural elucidation was performed using different spectroscopic techniques. The *in vitro* cytotoxic activity of all isolated compounds were examined against five human cancer cell lines. The results showed that the saponin fraction had potent *in vitro* cytotoxic activity against the five human cancer cell lines and was particularly potent against the PC-3 and A-549 cell lines. In addition, compound 1 exhibited potent activity against A-549 and PC-3. Moreover, compound 2 showed the maximum activity against PC-3. These biological results together with the molecular modeling study suggested that the cytotoxic activity of these compounds and in particular, compound 2, could occur *via* the inhibition of the HER-2 enzyme.

## Conflicts of interest

The authors declare no conflict of interest.

## Supplementary Material

RA-010-D0RA02775H-s001
