# Supplementary material for: Antiproliferative activity of new pentacyclic triterpene and a saponin from Gladiolus segetum Ker-Gawl corms supported by molecular docking study
Source: RSC Adv. 2020 Jun 12;10(38):22730–41. doi: 10.1039/d0ra02775h (PMC9054649; doi:10.1039/d0ra02775h)
Supplement: RA-010-D0RA02775H-s001 [file RA-010-D0RA02775H-s001.pdf]

Electronic Supplementary Material (ESI) for RSC Advances.
This journal is © The Royal Society of Chemistry 2020

Antiproliferative Activity of New Pentacyclic Triterpene and a Saponin from *Gladiolus Segetum* Ker-Gawl Corms supported by Molecular Docking Study

Adel M. Abd El-kader[a,b≠], Basma Khalaf Mahmoud[c≠], Dina Hajjar[d], Mamdouh F. A. Mohamed[e], Alaa M. Hayallah[f,g], Usama Ramadan Abdelmohsen[a,c,h] *

[a] *Department of Pharmacognosy, Faculty of Pharmacy, Deraya University, Minia, Egypt*
[b]*Department of Pharmacognosy, Faculty of Pharmacy, Al-Azhar University, Assiut 71524, Egypt*
[c]*Department of Pharmacognosy, Faculty of Pharmacy, Minia University, 61519 Minia, Egypt*
[d]*Department of Biochemistry, Collage of Science, University of Jeddah, 80203 Jeddah, Saudi Arabia*
[e]*Department of Pharmaceutical Chemistry, Faculty of Pharmacy, Sohag University, 82524 Sohag, Egypt.*
[f]*Pharmaceutical Chemistry Department, Faculty of pharmacy, Deraya University, Minia, Egypt*
[g]*Department of Pharmaceutical Organic Chemistry, Faculty of Pharmacy, Assiut University, Assiut 71526, Egypt*
[h]*Department of Pharmacognosy, College of Pharmacy, King Khalid University, Abha 61441, Saudi Arabia*

≠ *Those authors have equally contributed to this work*

To whom correspondence should be addressed.

Usama Ramadan Abdelmohsen, **Tel.:** +2-86-2347759, **E-mail**: usama.ramadan@mu.edu.eg

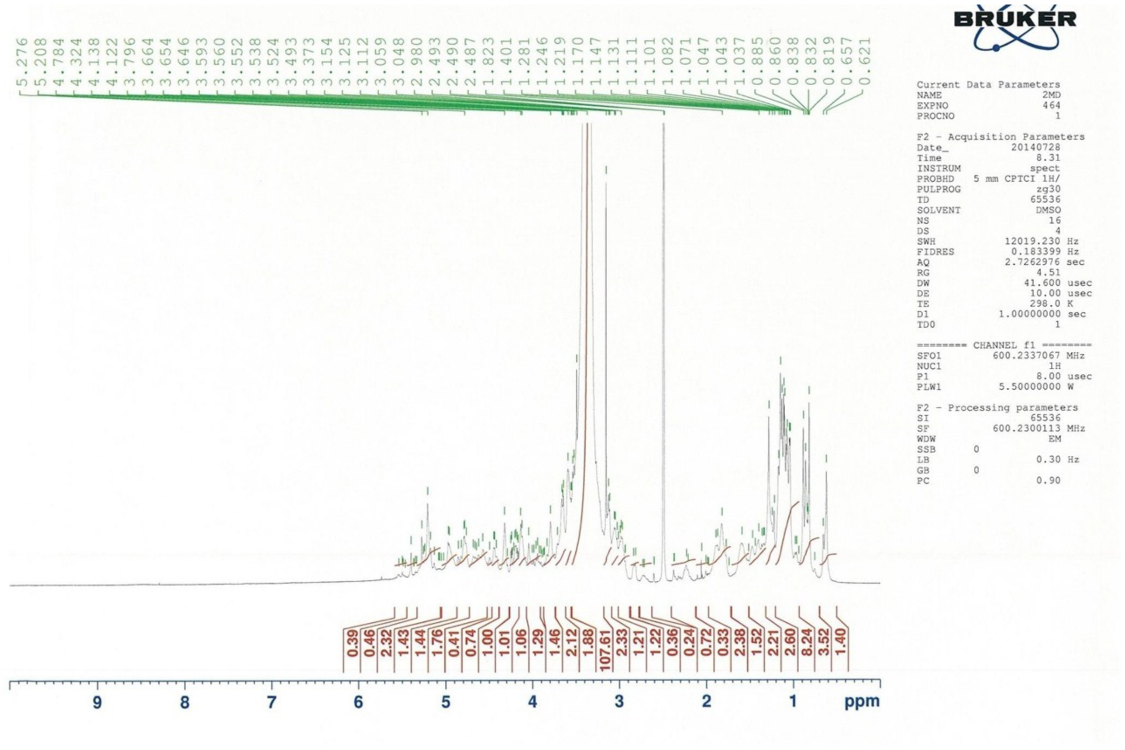

Fig. 1S. ^{1}H-NMR spectrum of compound 1 (600 MHz, DMSO-d_6)

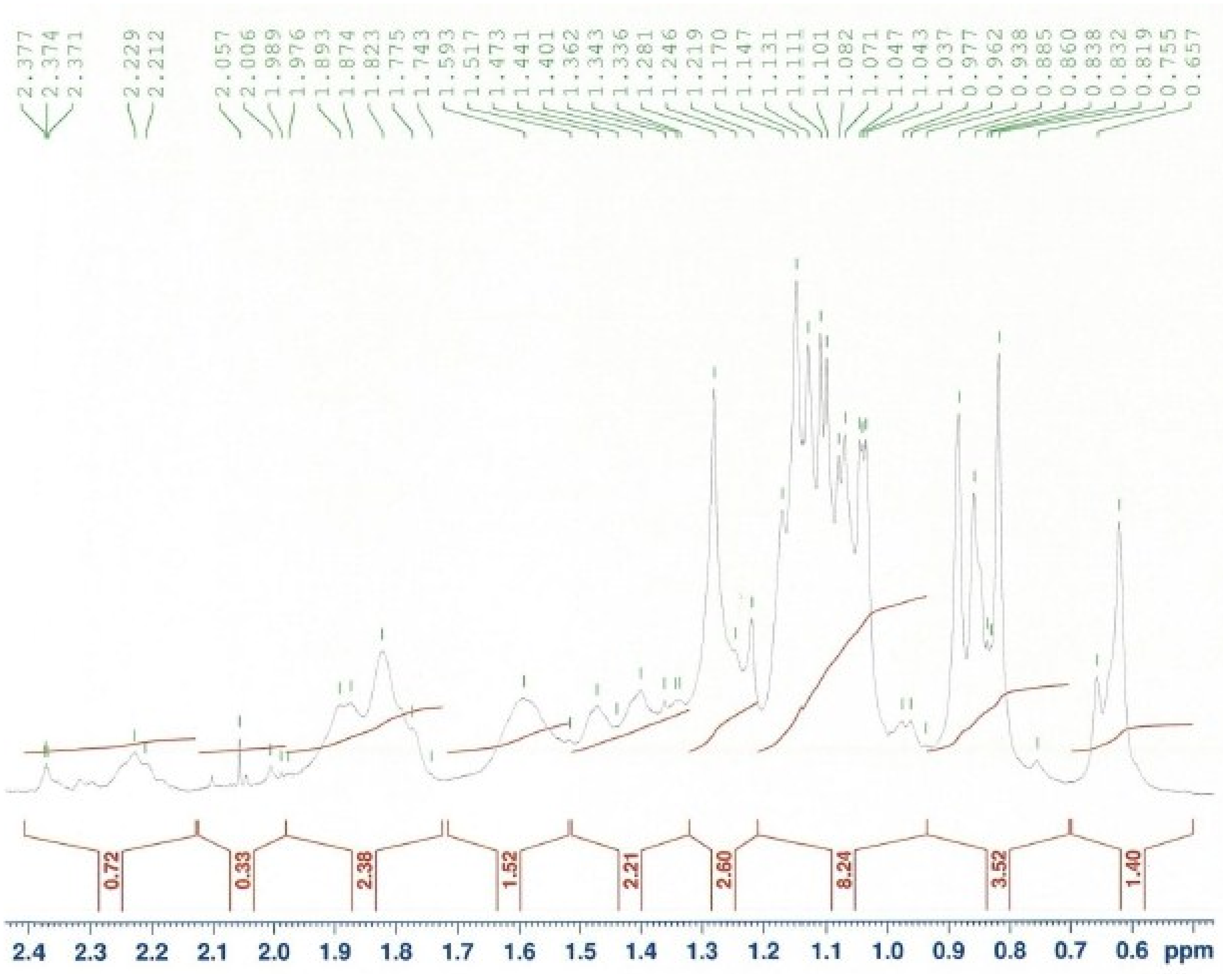

Fig. 2S. Expanded ^{1}H-NMR spectrum of compound 1 (600 MHz, DMSO-d_6)

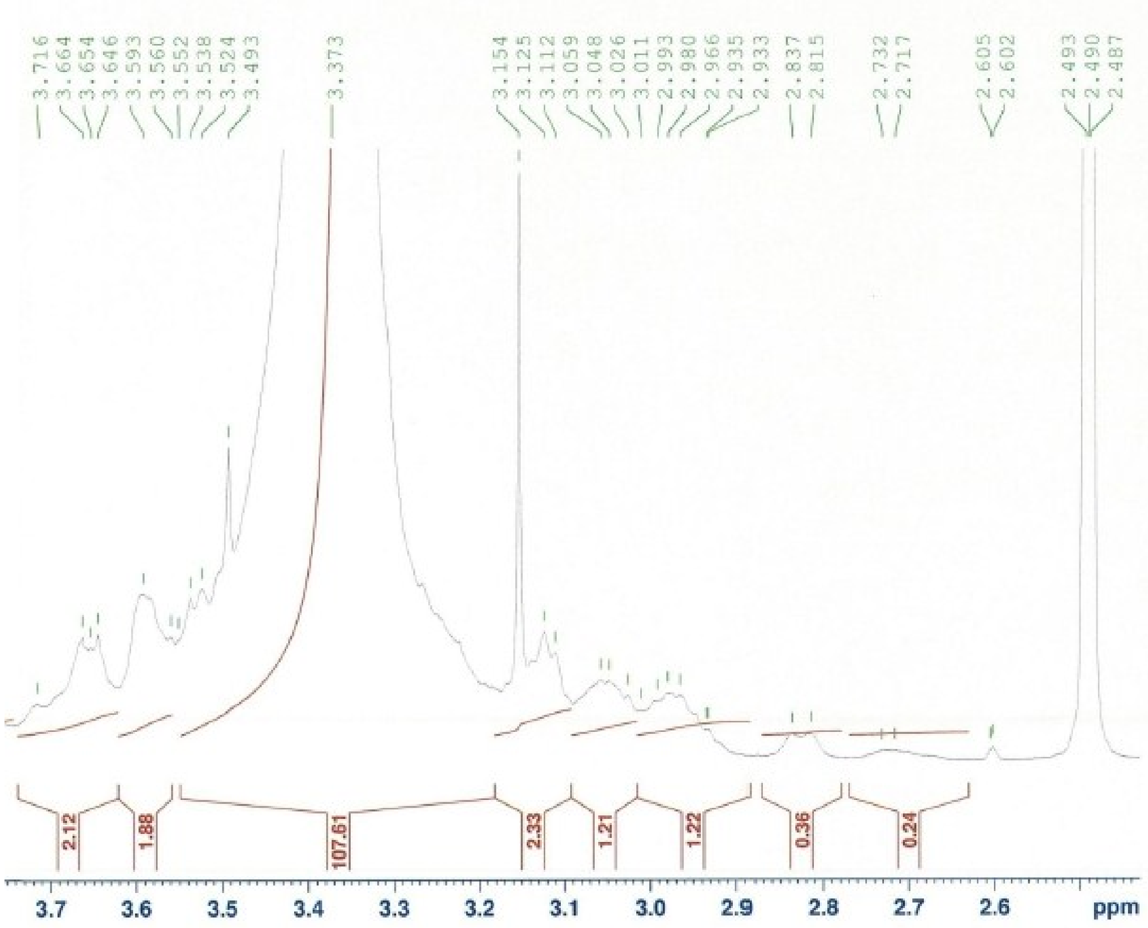

Fig. 3S. Expanded ^{1}H-NMR spectrum of compound 1 (600 MHz, DMSO-d_6)

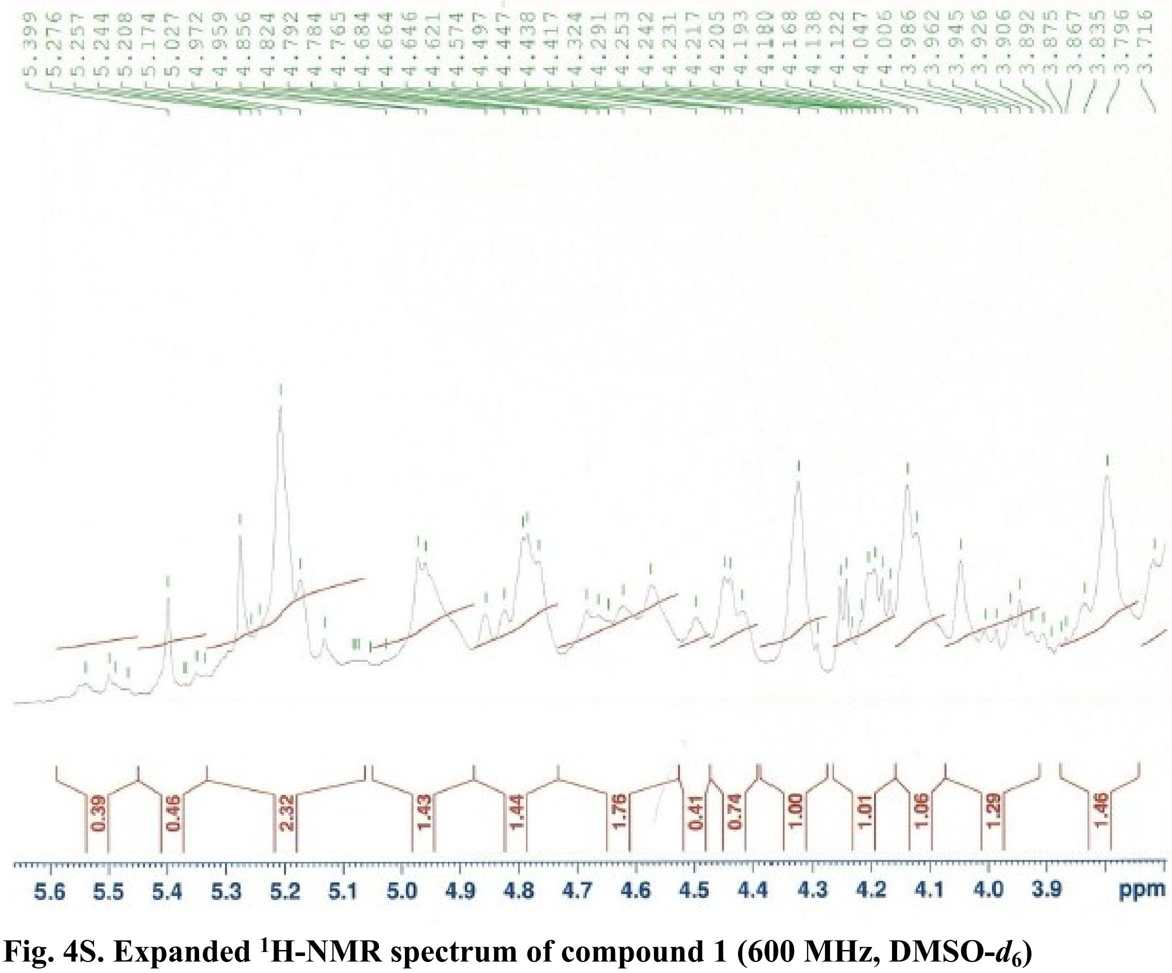

Fig. 4S. Expanded ^{1}H-NMR spectrum of compound 1 (600 MHz, DMSO-d_6)

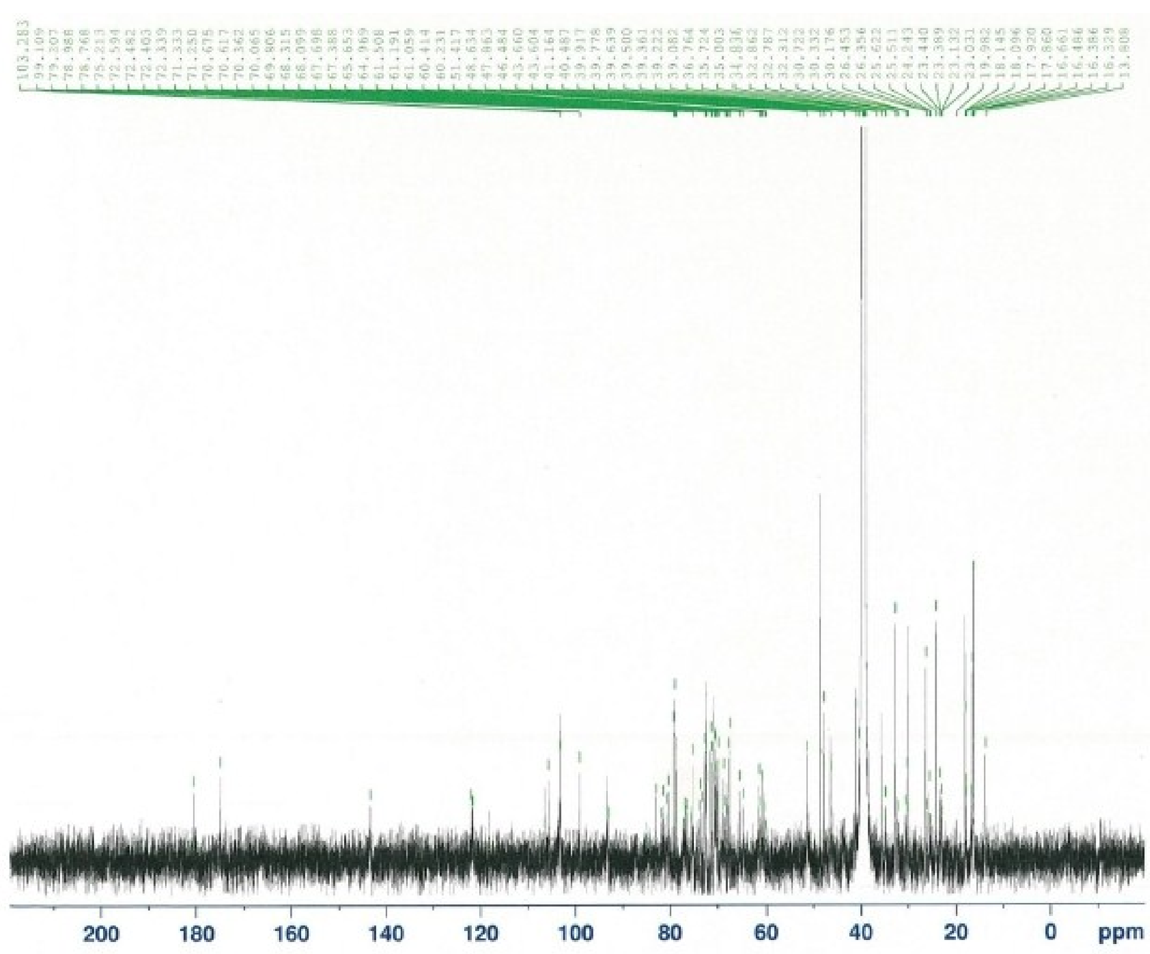

Fig. 5S. ^{13}C-NMR spectrum of compound 1 (150 MHz, DMSO-d_6)

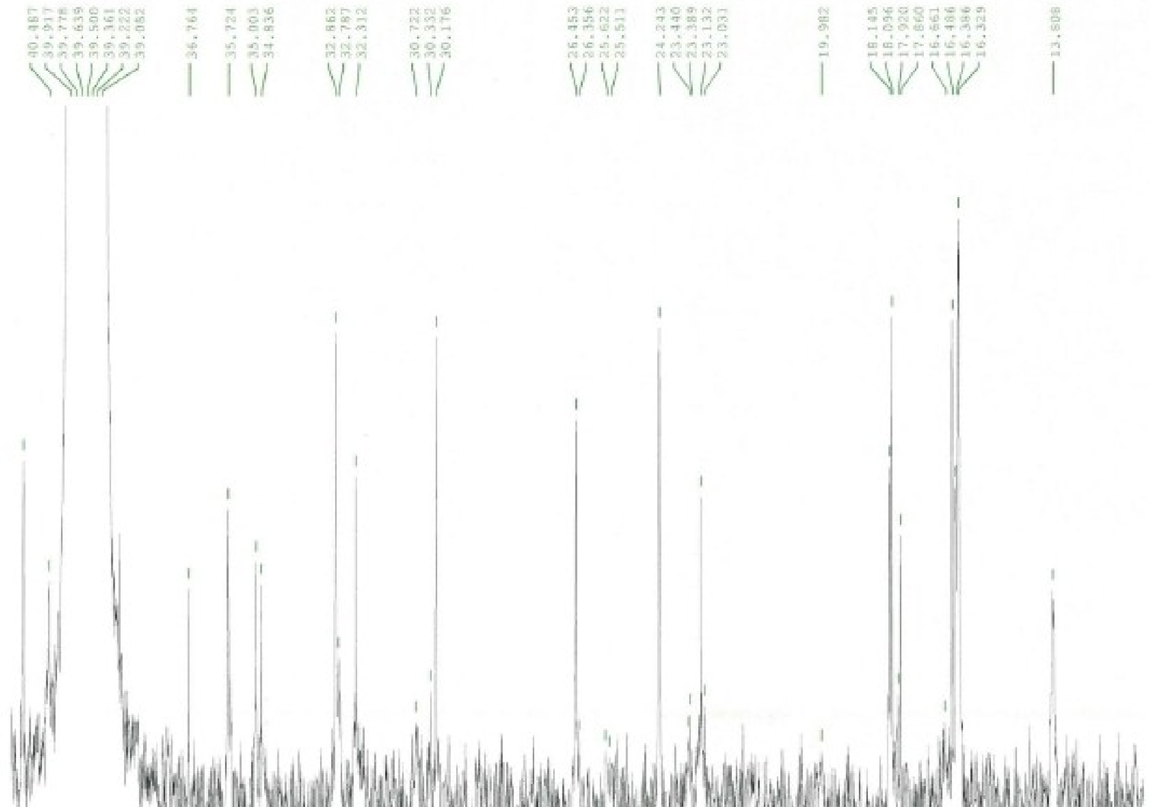

Fig. 6S.Expanded ^{13}C-NMR spectrum of compound 1 (150 MHz, DMSO-d_6)

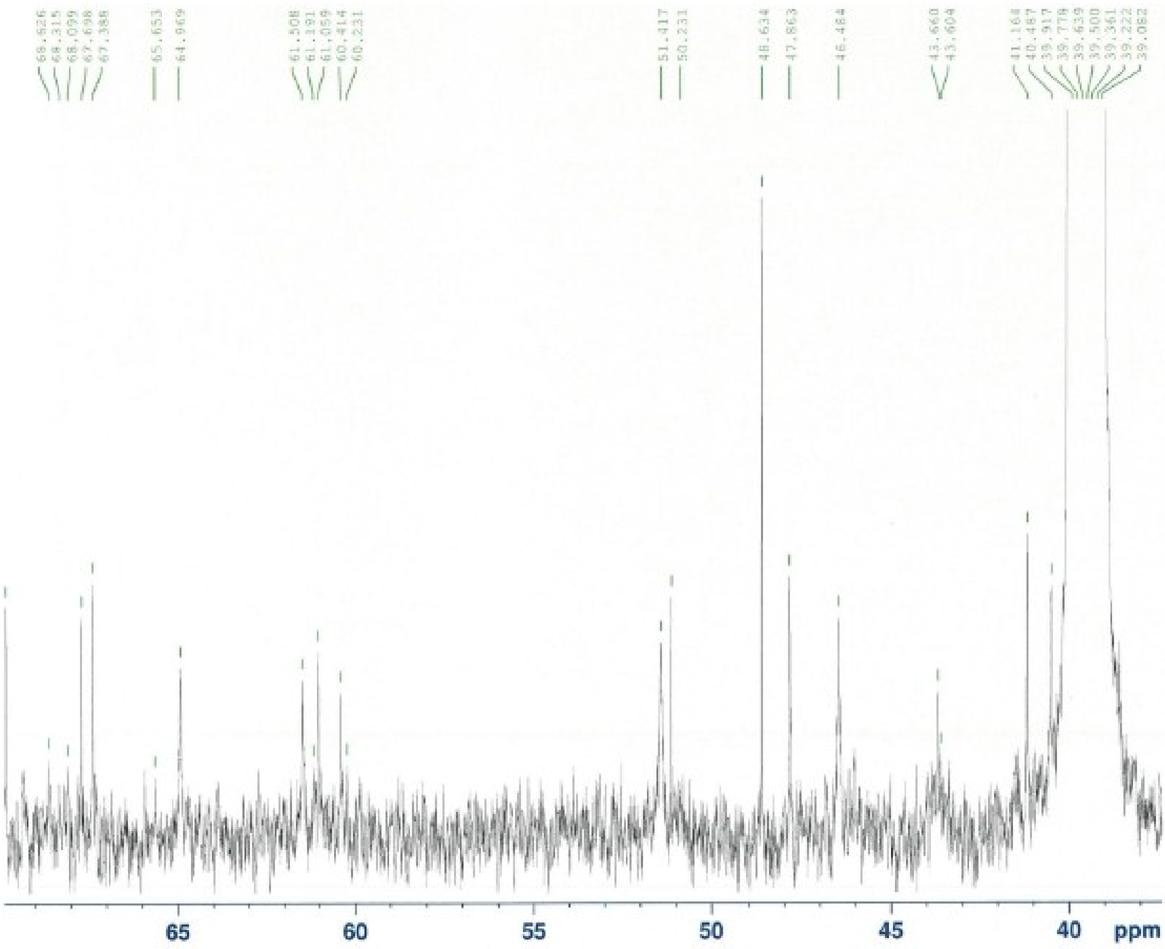

Fig. 7S.Expanded ^{13}C-NMR spectrum of compound 1 (150 MHz, DMSO-d_6)

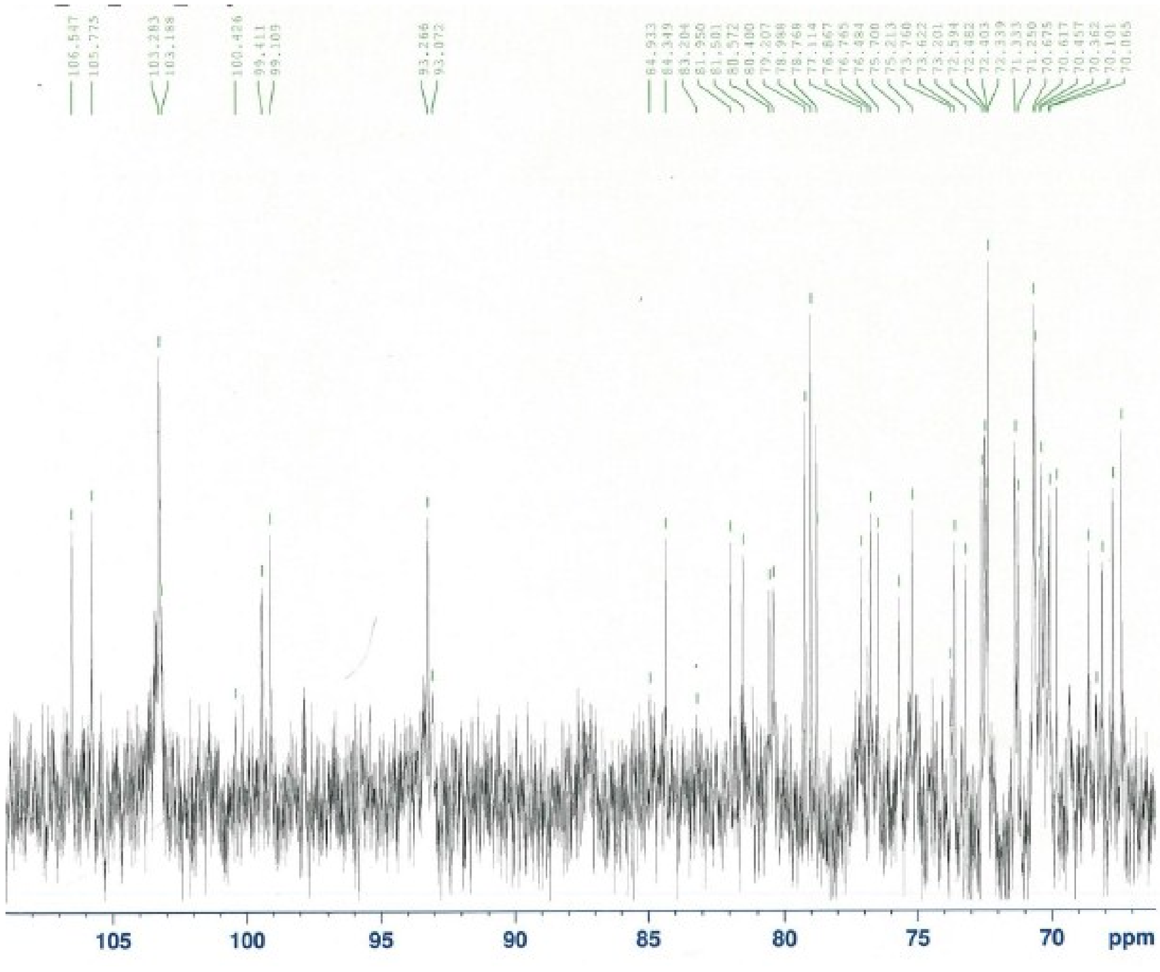

Fig. 8S.Expanded ^{13}C-NMR spectrum of compound 1 (150 MHz, DMSO-d_6)

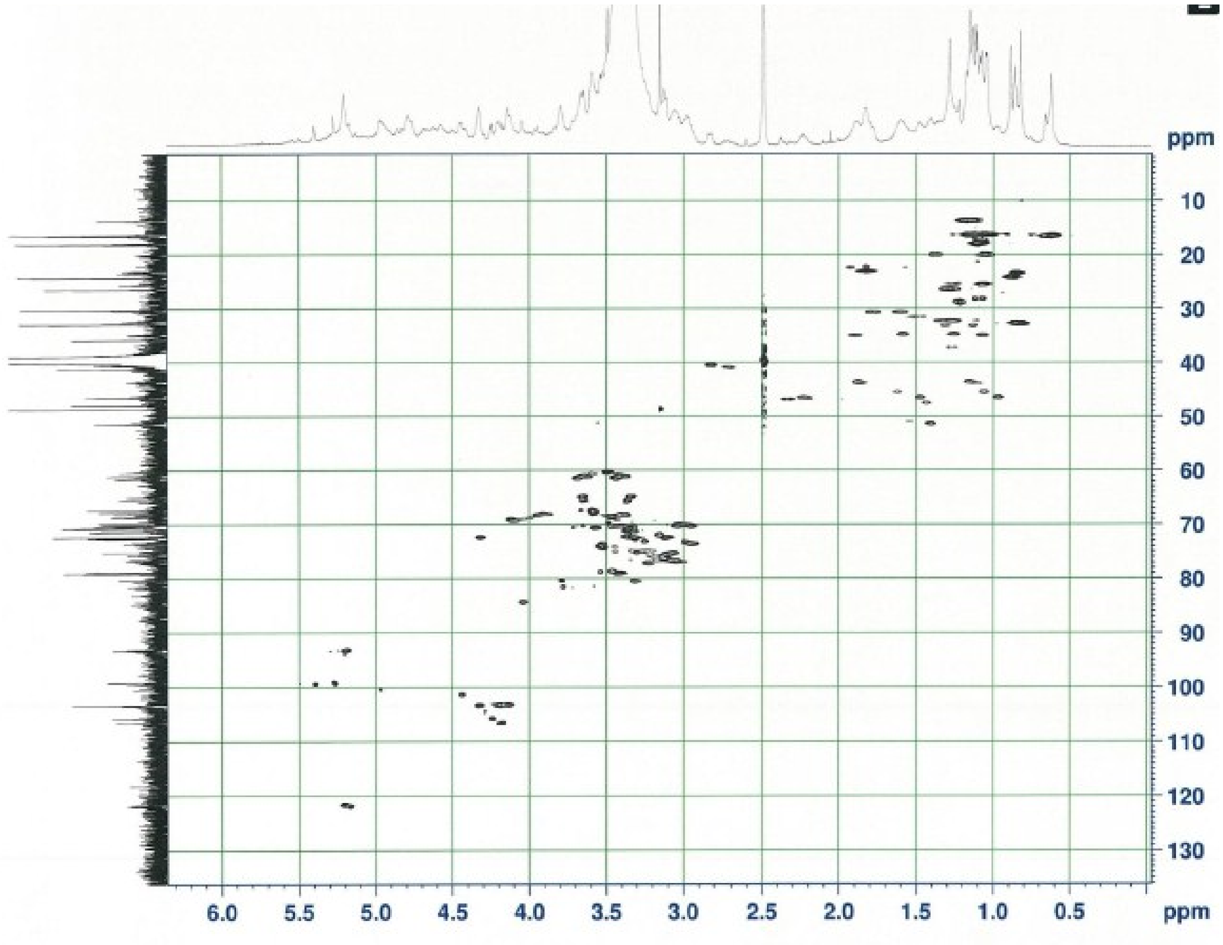

Fig. 9S. HSQC spectrum of compound 1

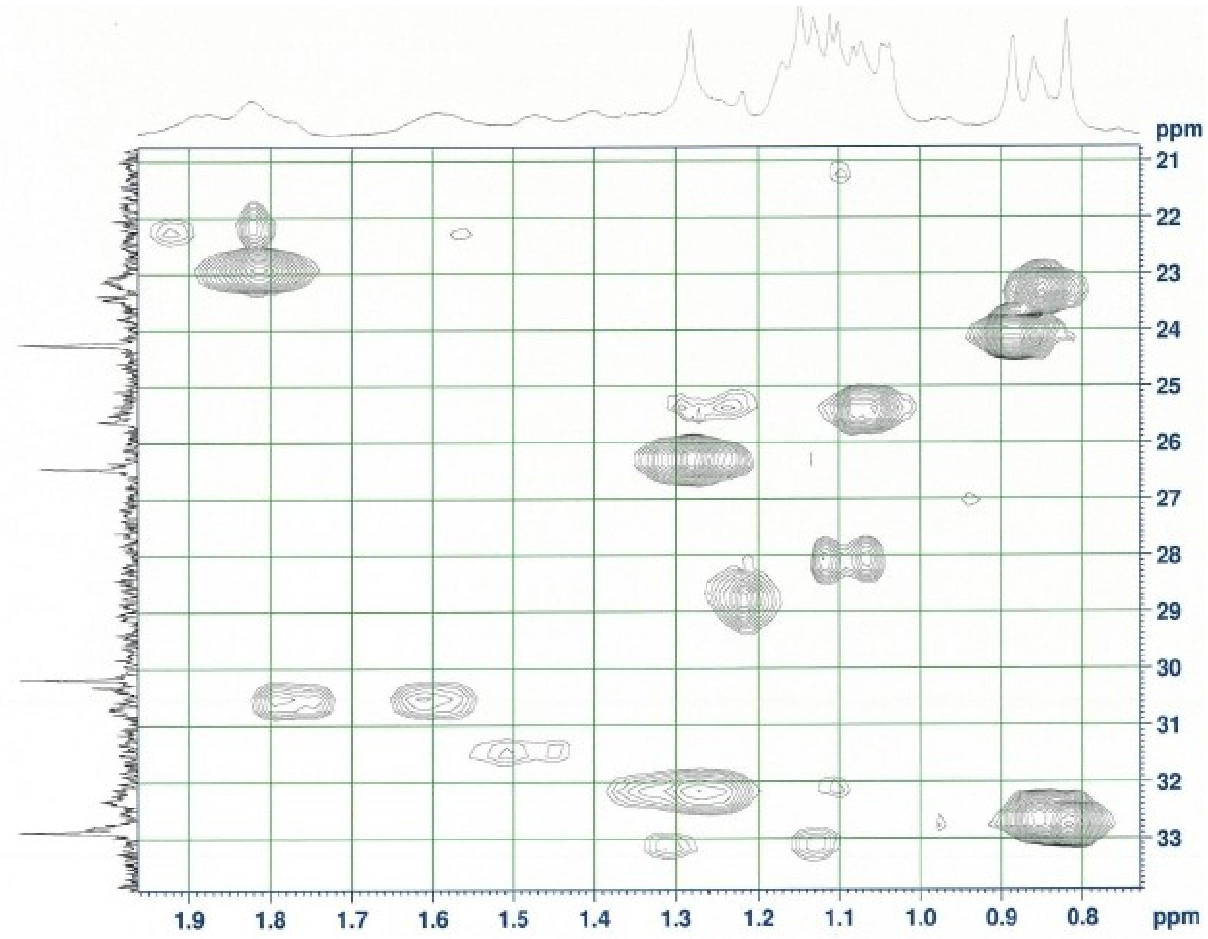

Fig. 10S. Expanded HSQC spectrum of compound 1

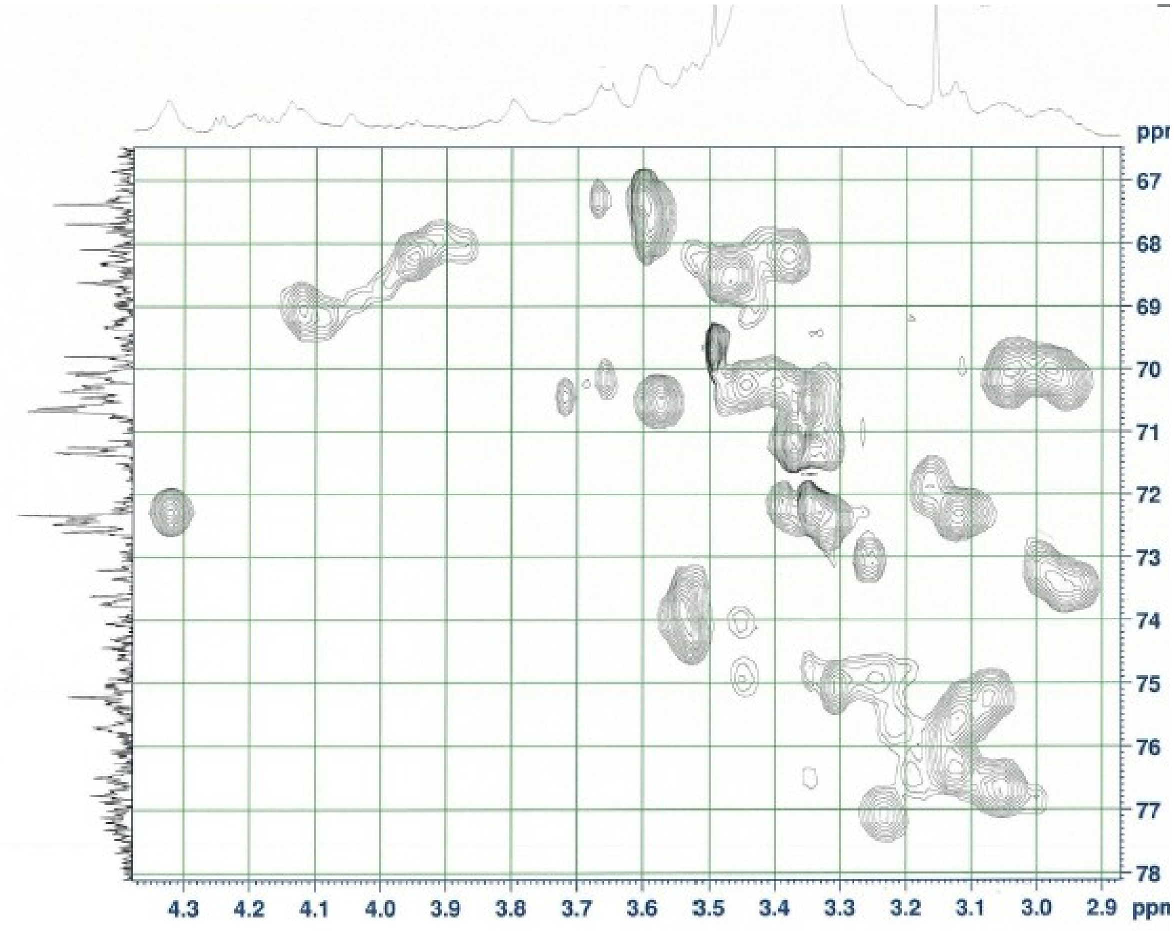

Fig. 11S. Expanded HSQC spectrum of compound 1

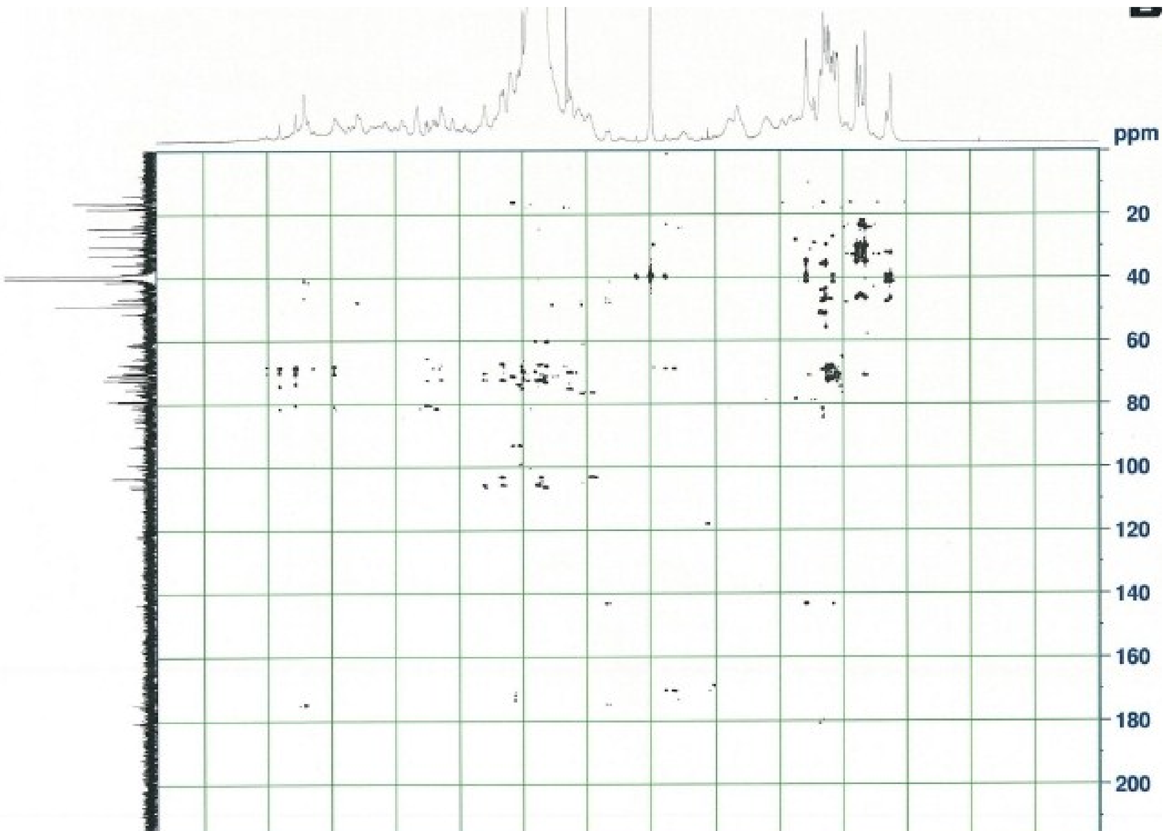

Fig. 12S. HMBC spectrum of compound 1

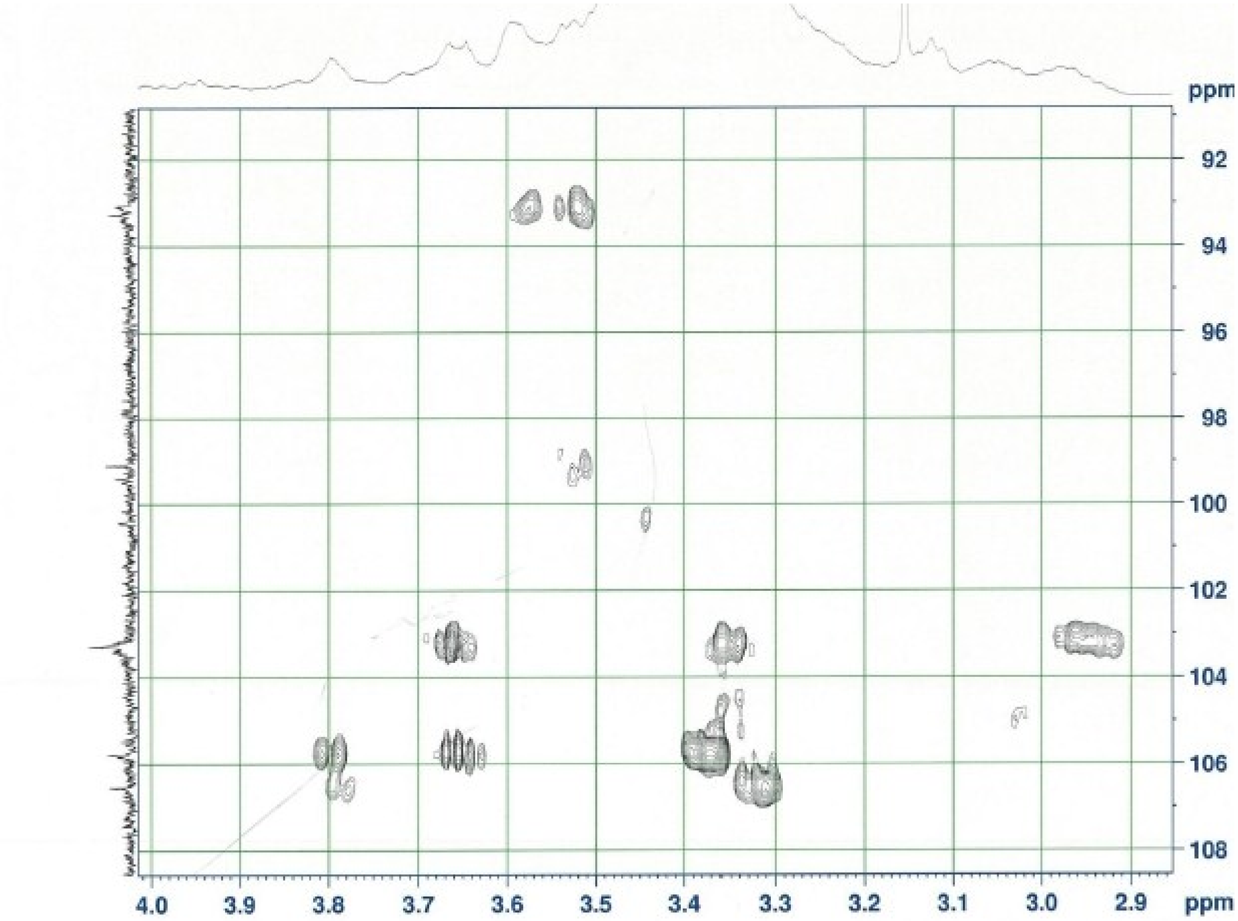

Fig. 13S. Expanded HMBC spectrum of compound 1

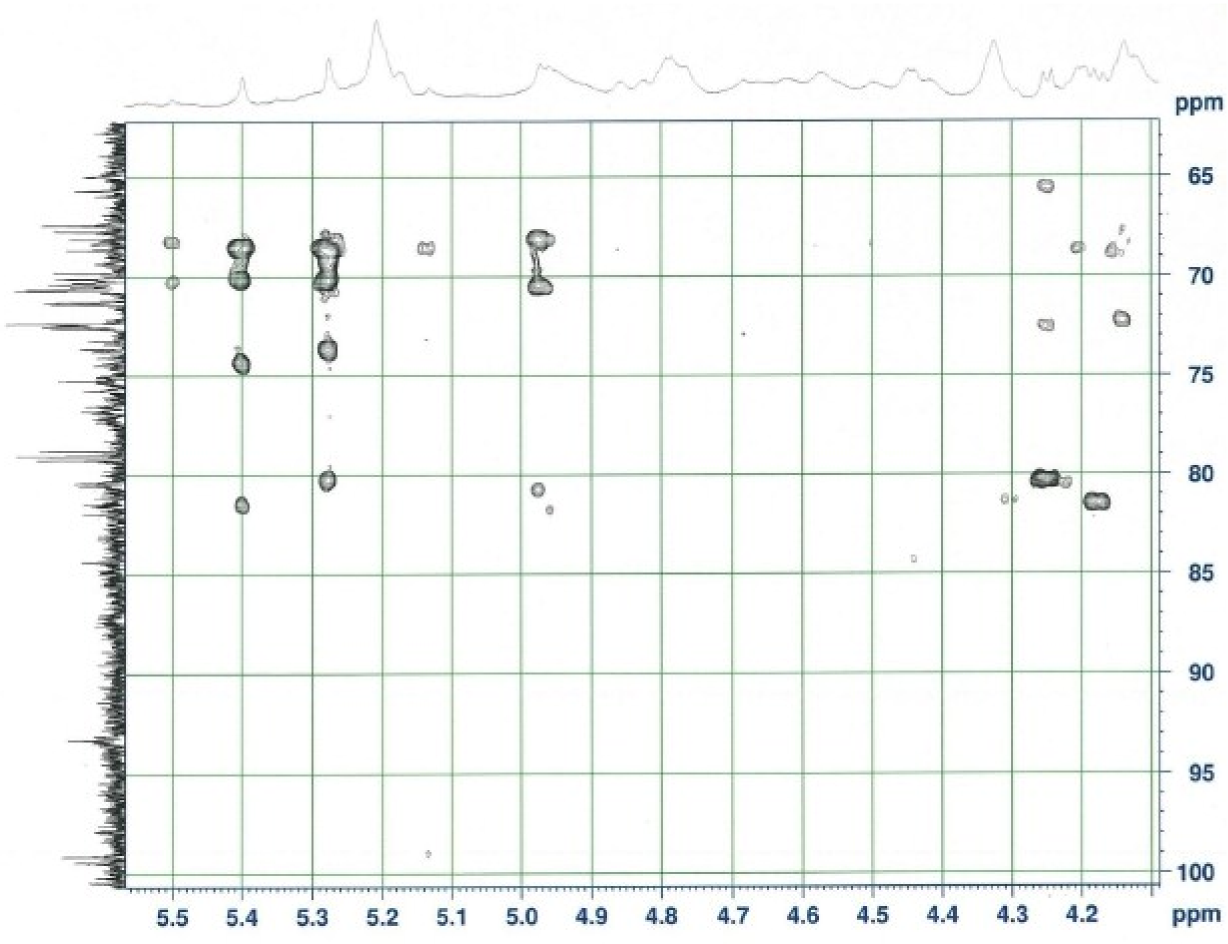

Fig. 14S. Expanded HMBC spectrum of compound 1

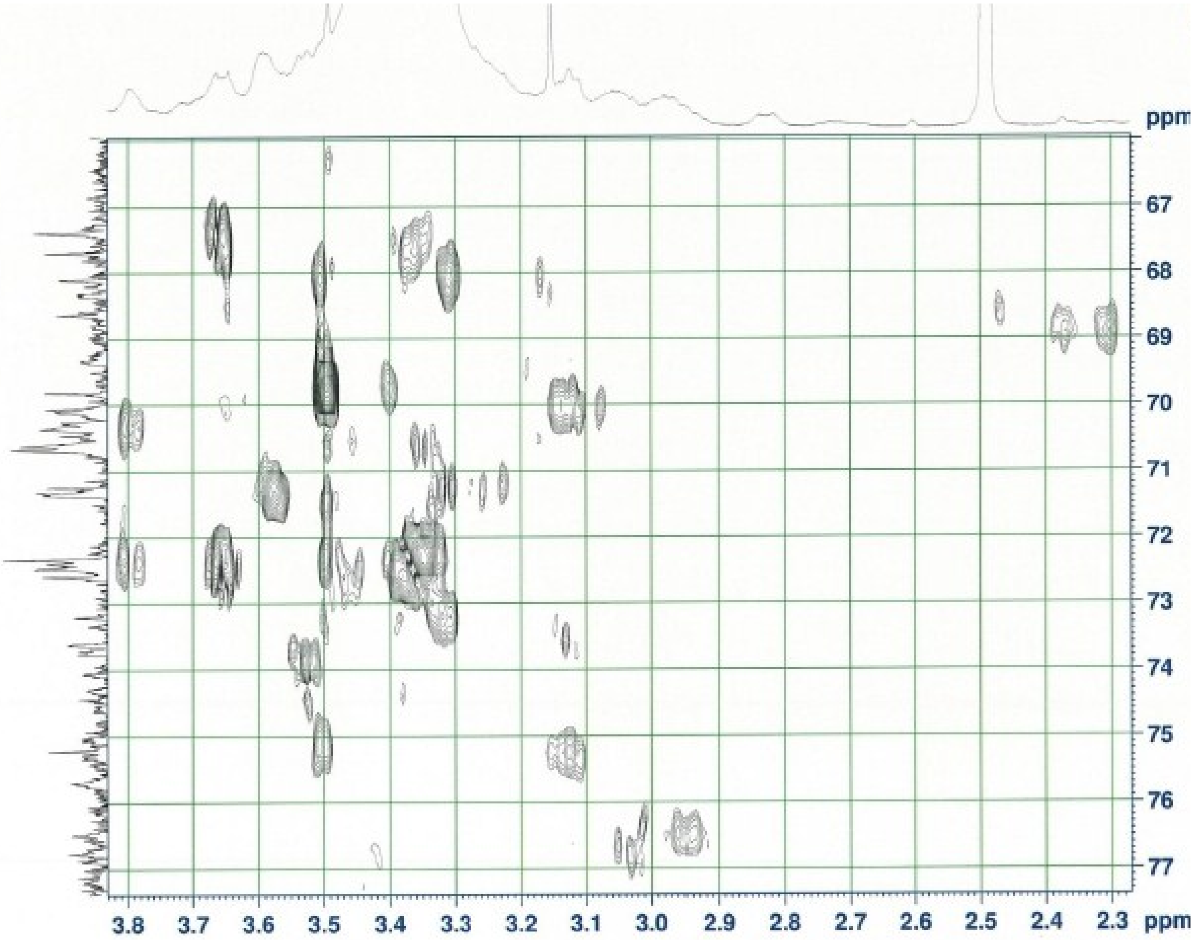

Fig. 15S. Expanded HMBC spectrum of compound 1

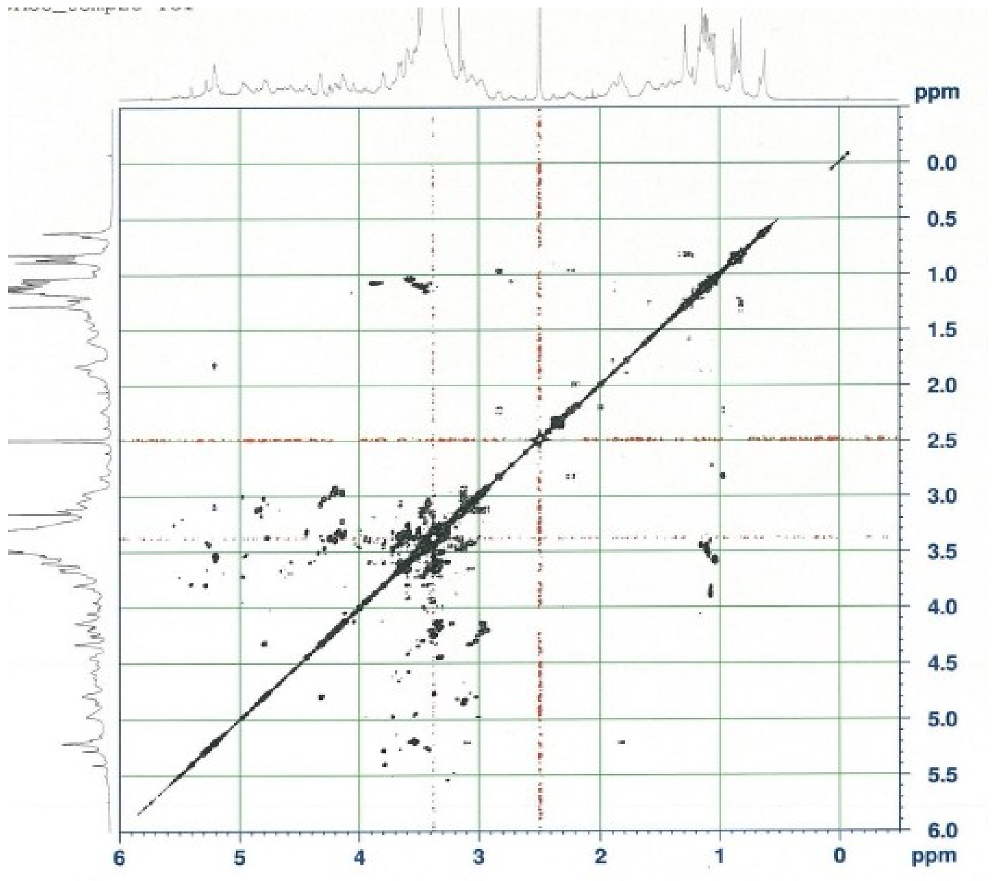

Fig. 16S. ^{1}H-^{1}H COSY spectrum of compound 1

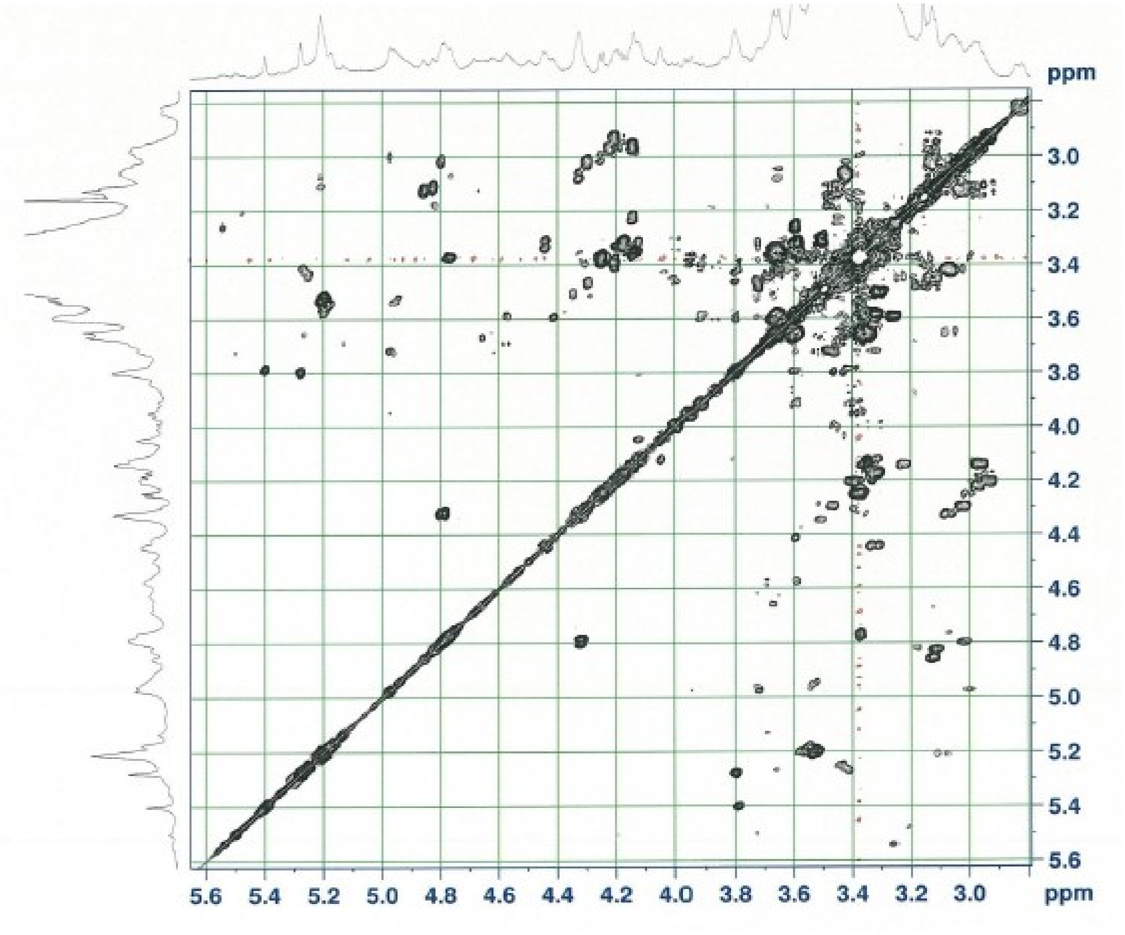

Fig. 17S.Expanded ^{1}H-^{1}H COSY spectrum of compound 1

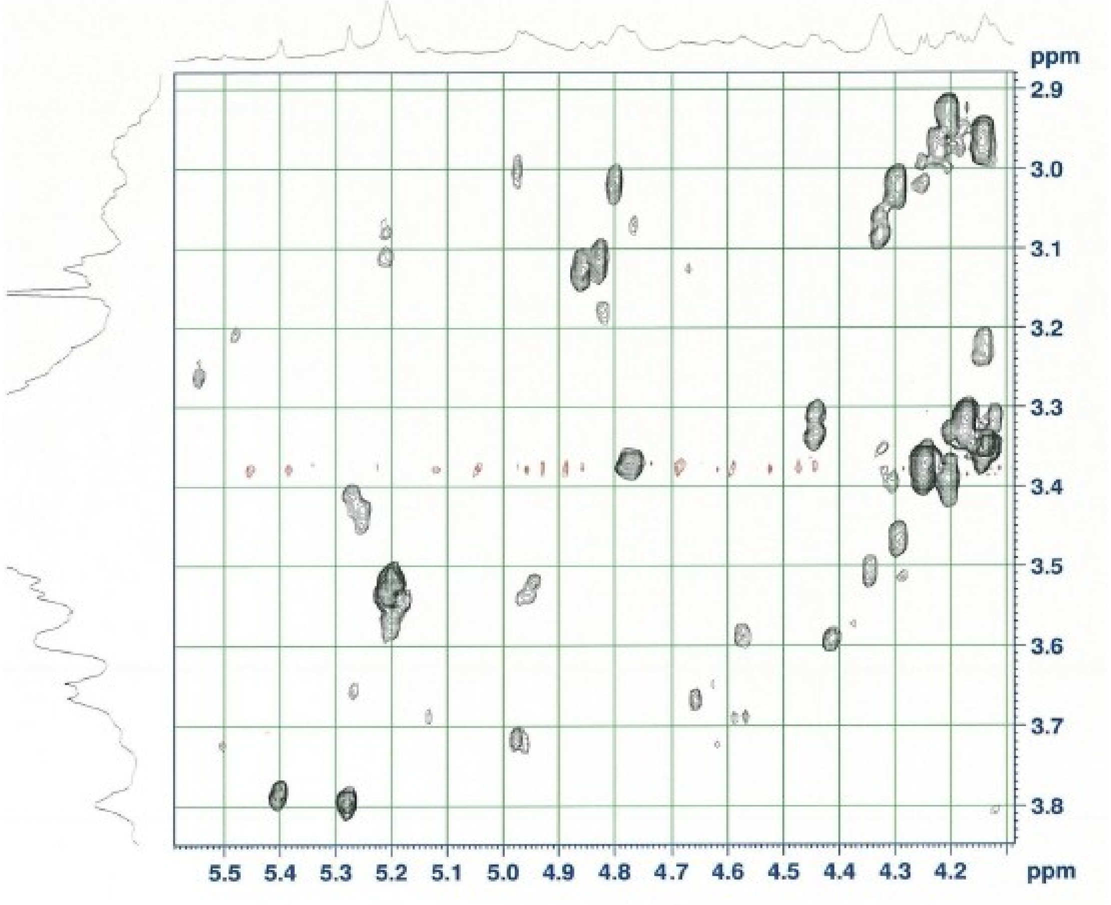

Fig. 18S.Expanded ^{1}H-^{1}H COSY spectrum of compound 1

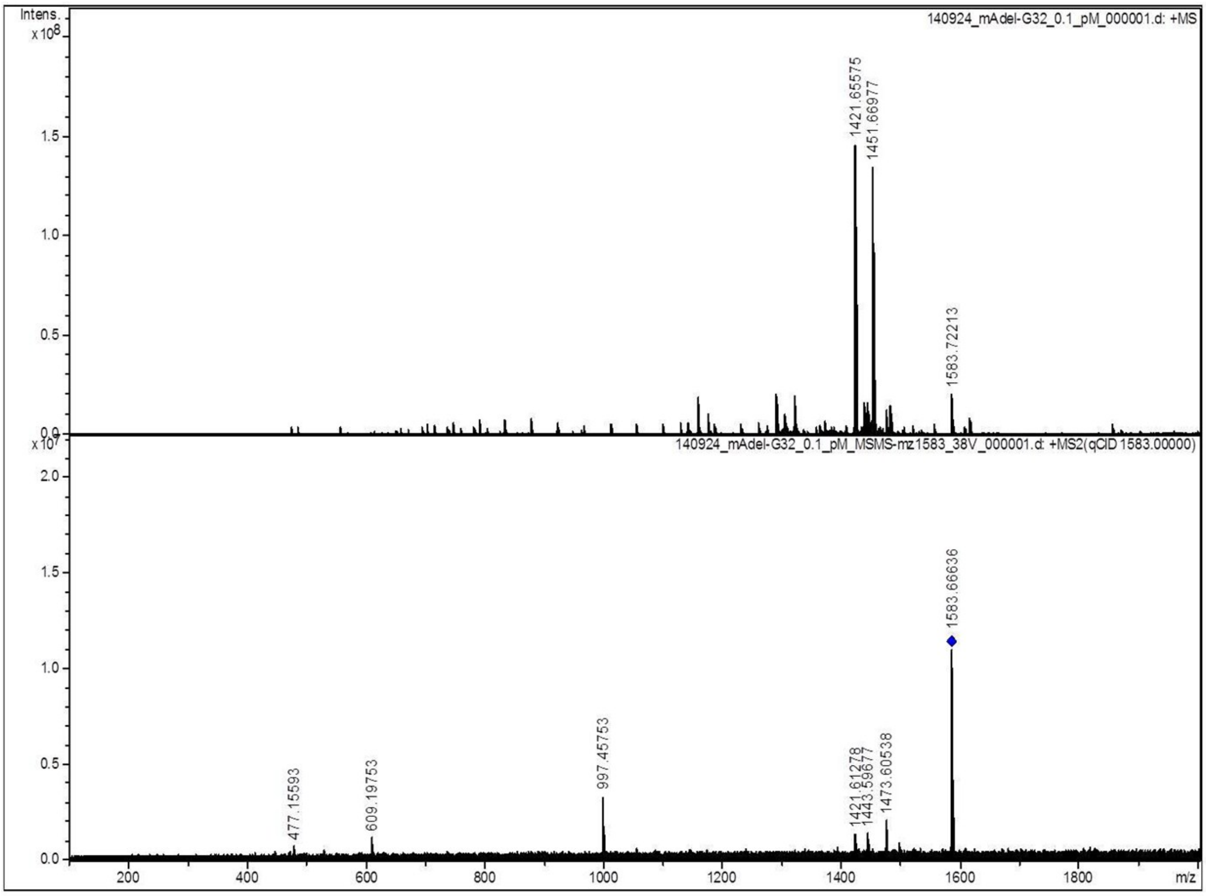

Fig. 19S. HR-ESI-MS spectrum of compound 1

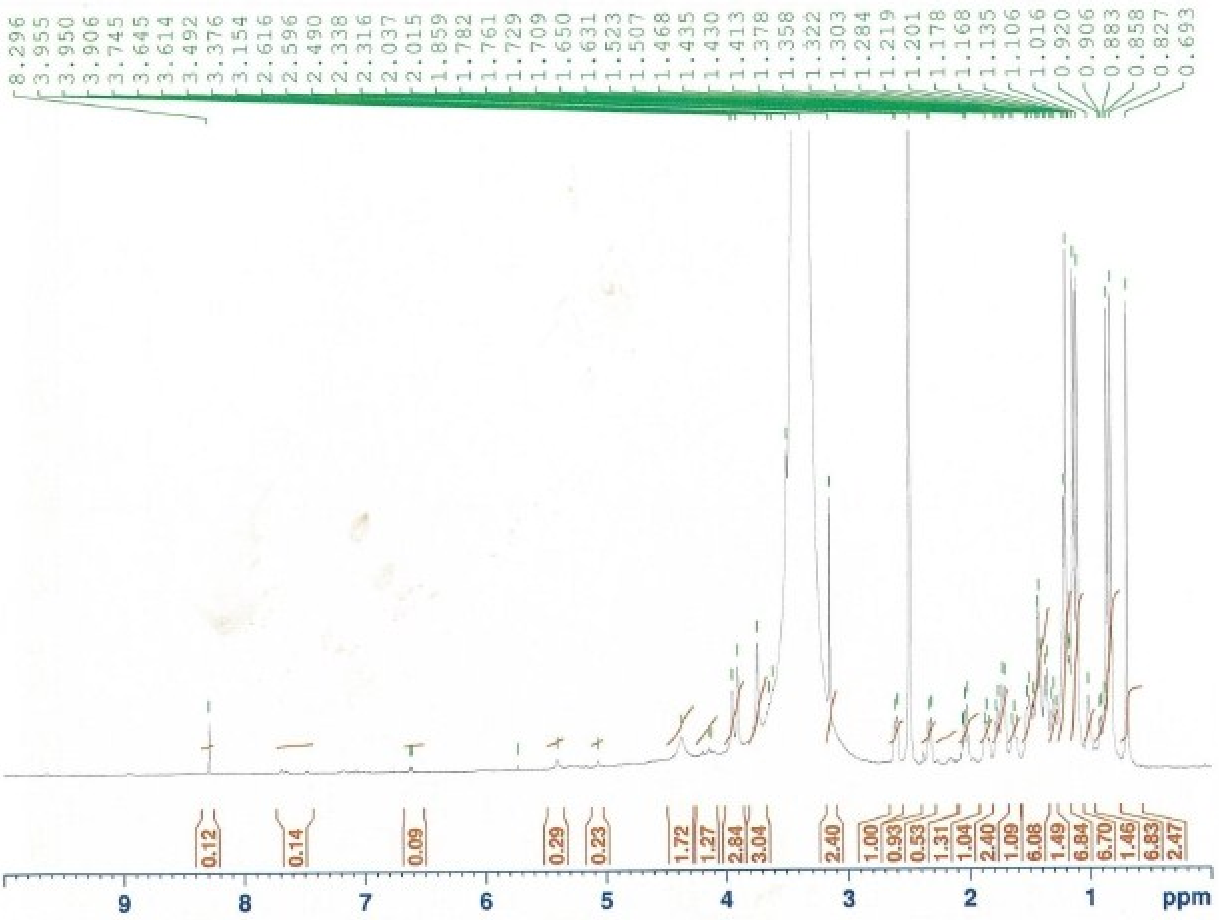

Fig. 20S. ^{1}H-NMR spectrum of compound 2 (600 MHz, DMSO-d_6)

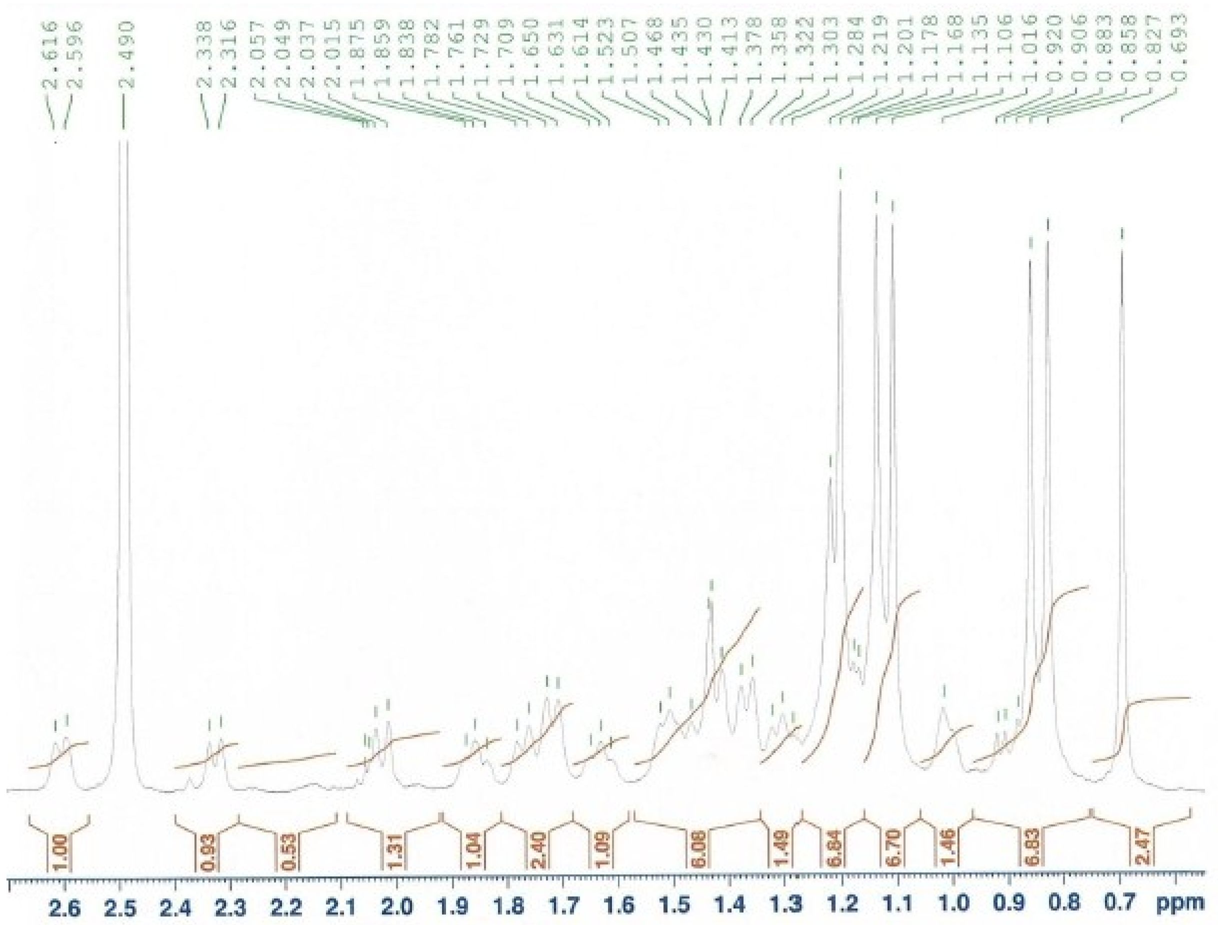

Fig. 21S. Expanded ^{1}H-NMR spectrum of compound 2 (600 MHz, DMSO-d_6)

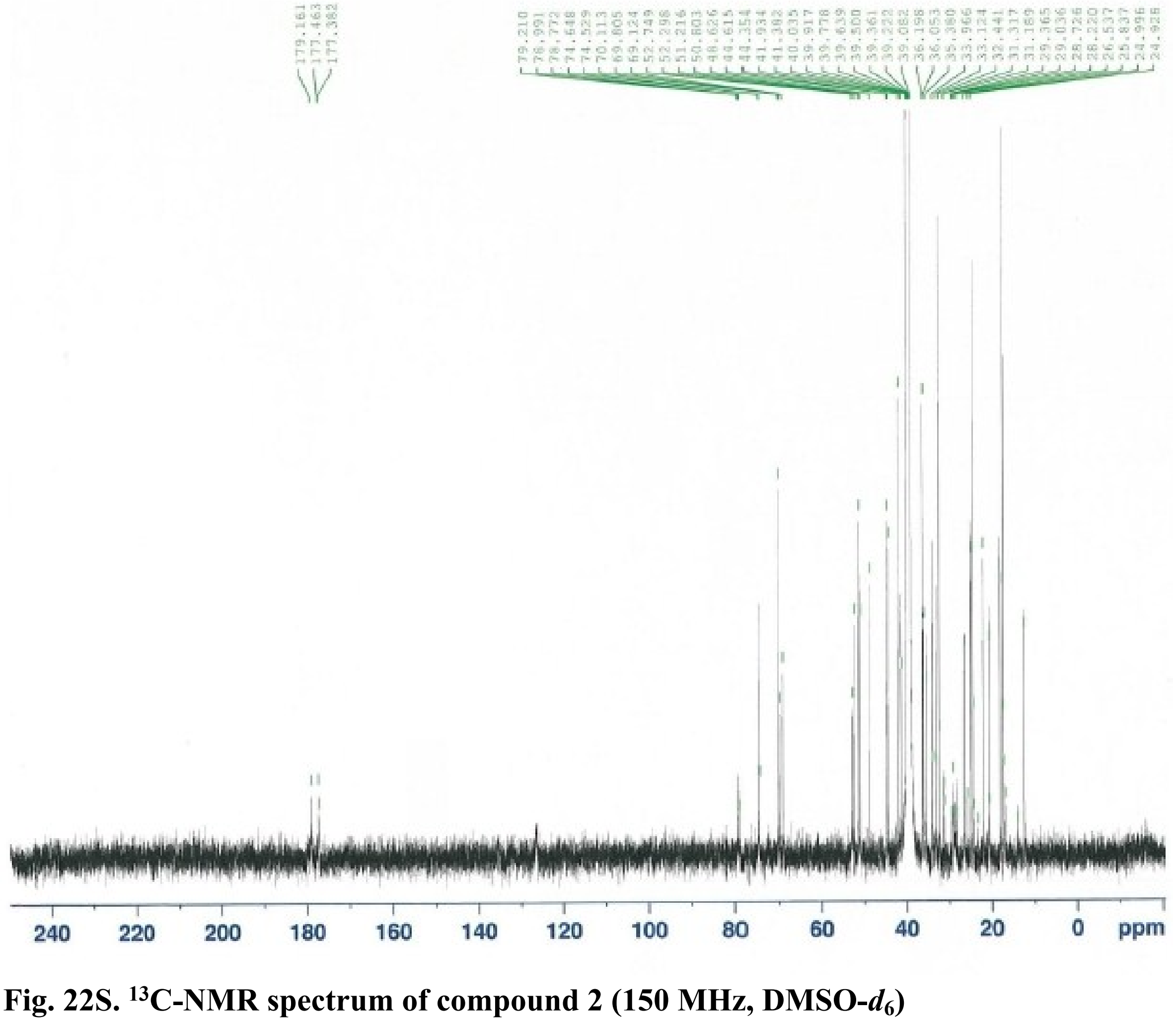

Fig. 22S. ^{13}C-NMR spectrum of compound 2 (150 MHz, DMSO-d_6)

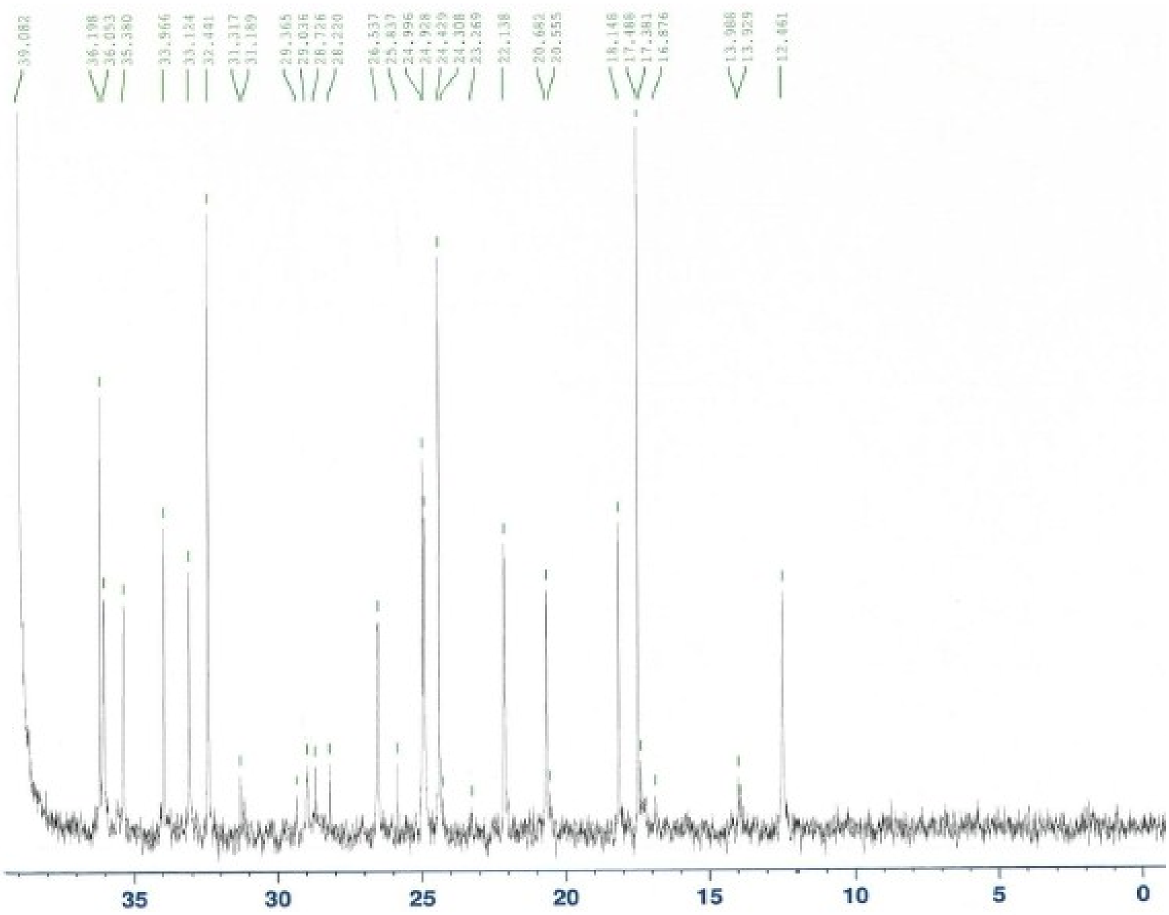

Fig. 23S. Expanded ^{13}C-NMR spectrum of compound 2 (150 MHz, DMSO-d_6)

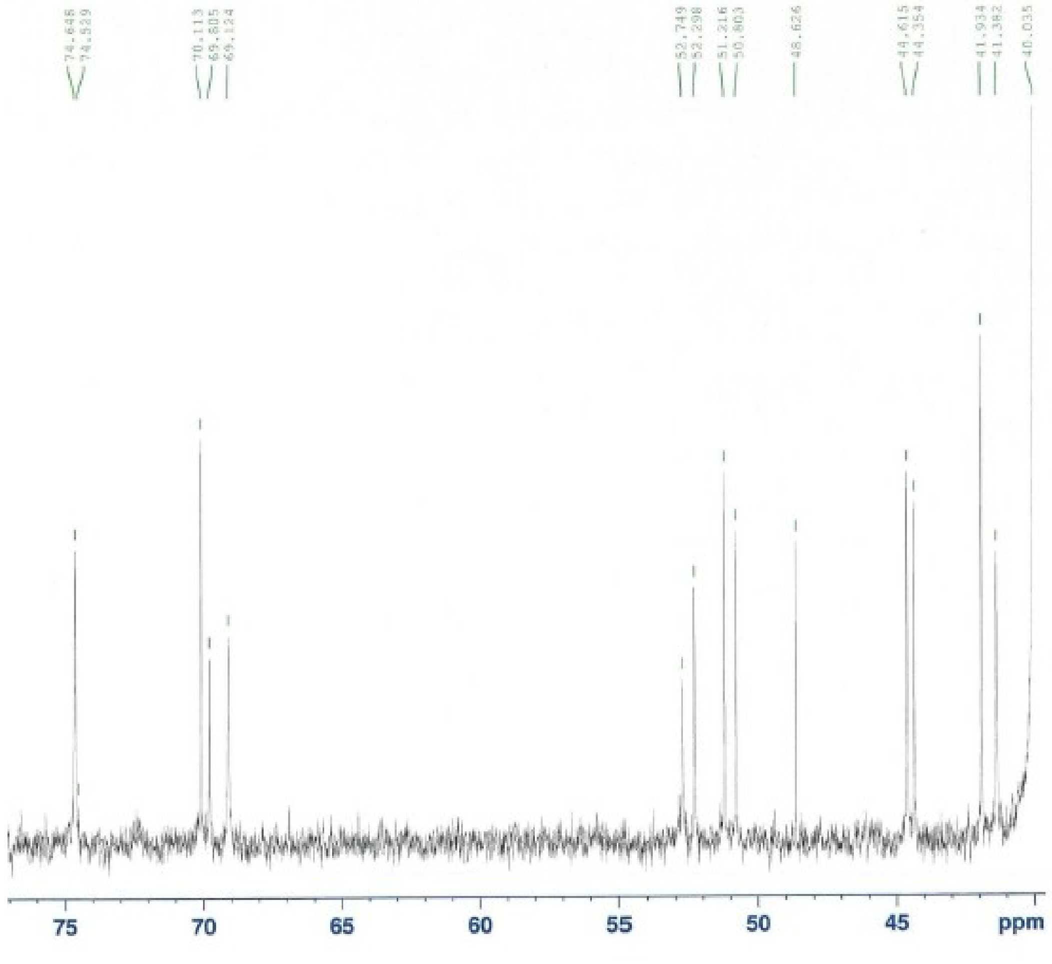

Fig. 24S. Expanded ^{13}C-NMR spectrum of compound 2 (150 MHz, DMSO-d_6)

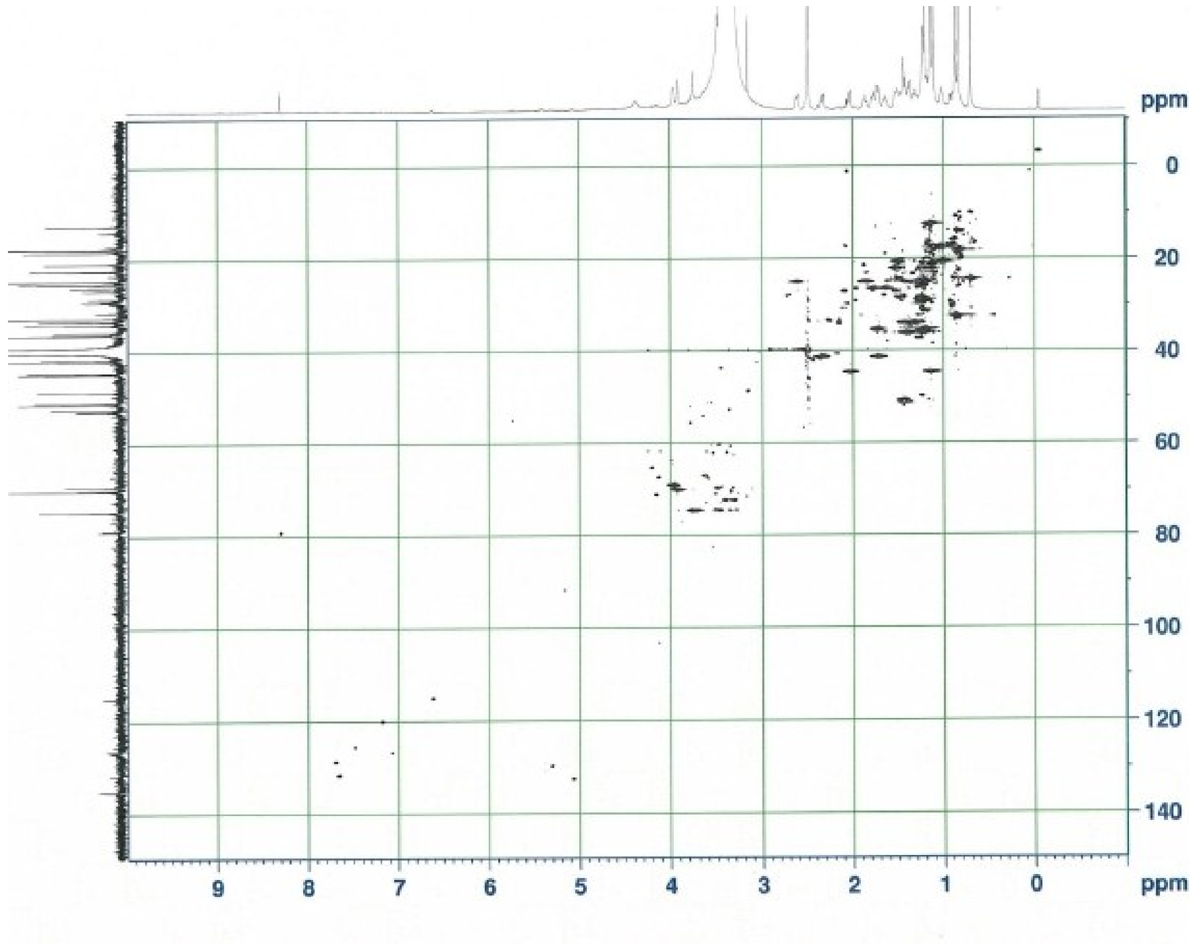

Fig. 25S. HSQC spectrum of compound 2

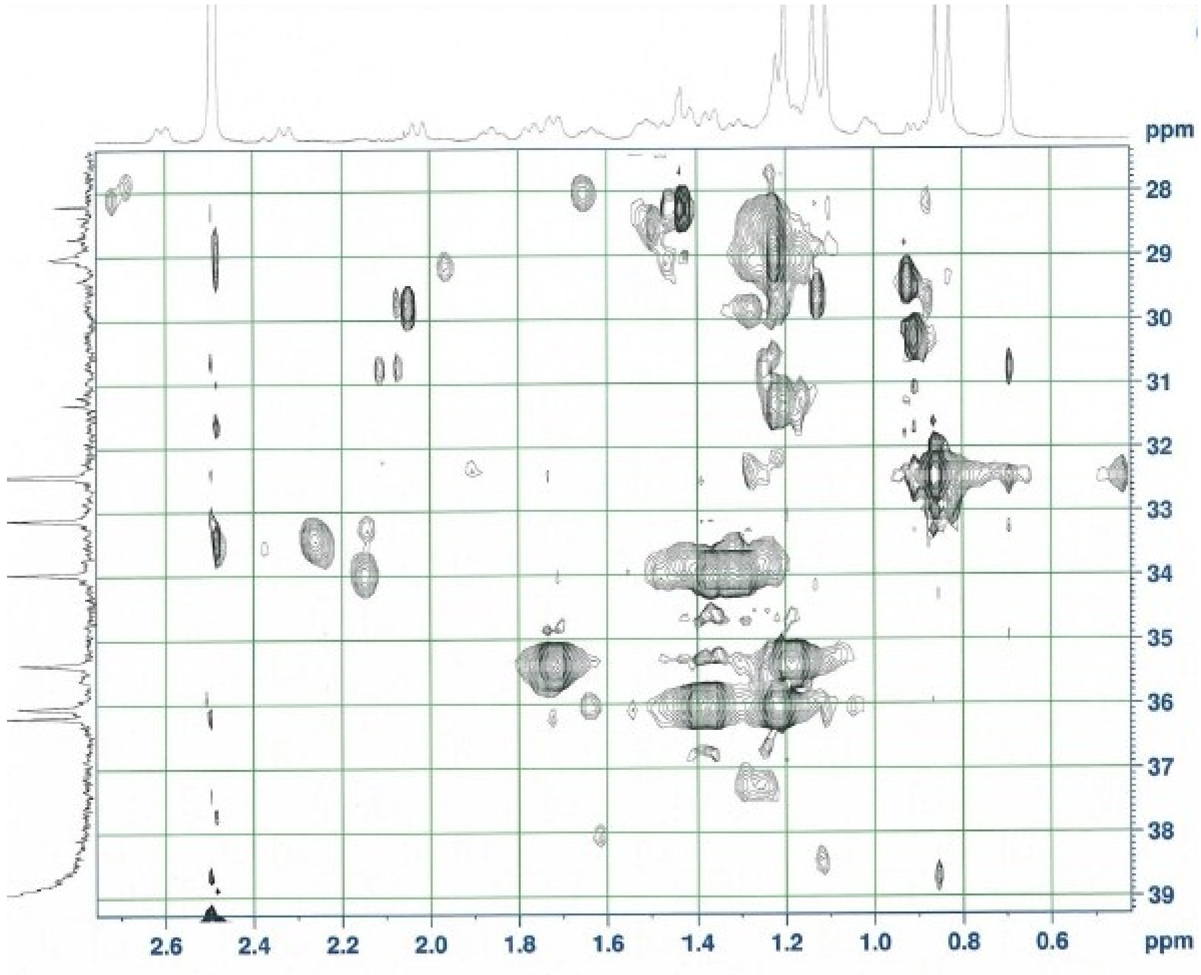

Fig. 26S. Expnaded HSQC spectrum of compound 2

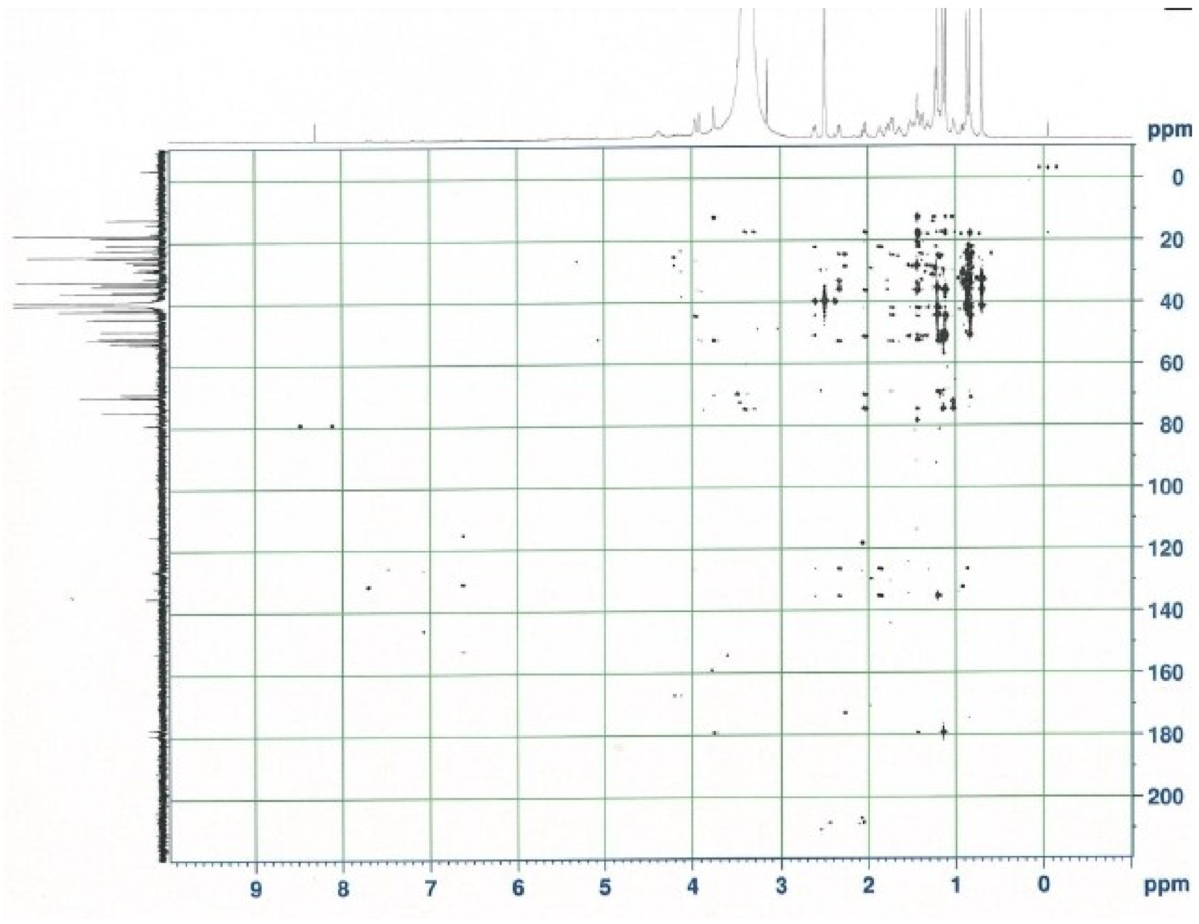

Fig. 27S. HMBC spectrum of compound 2

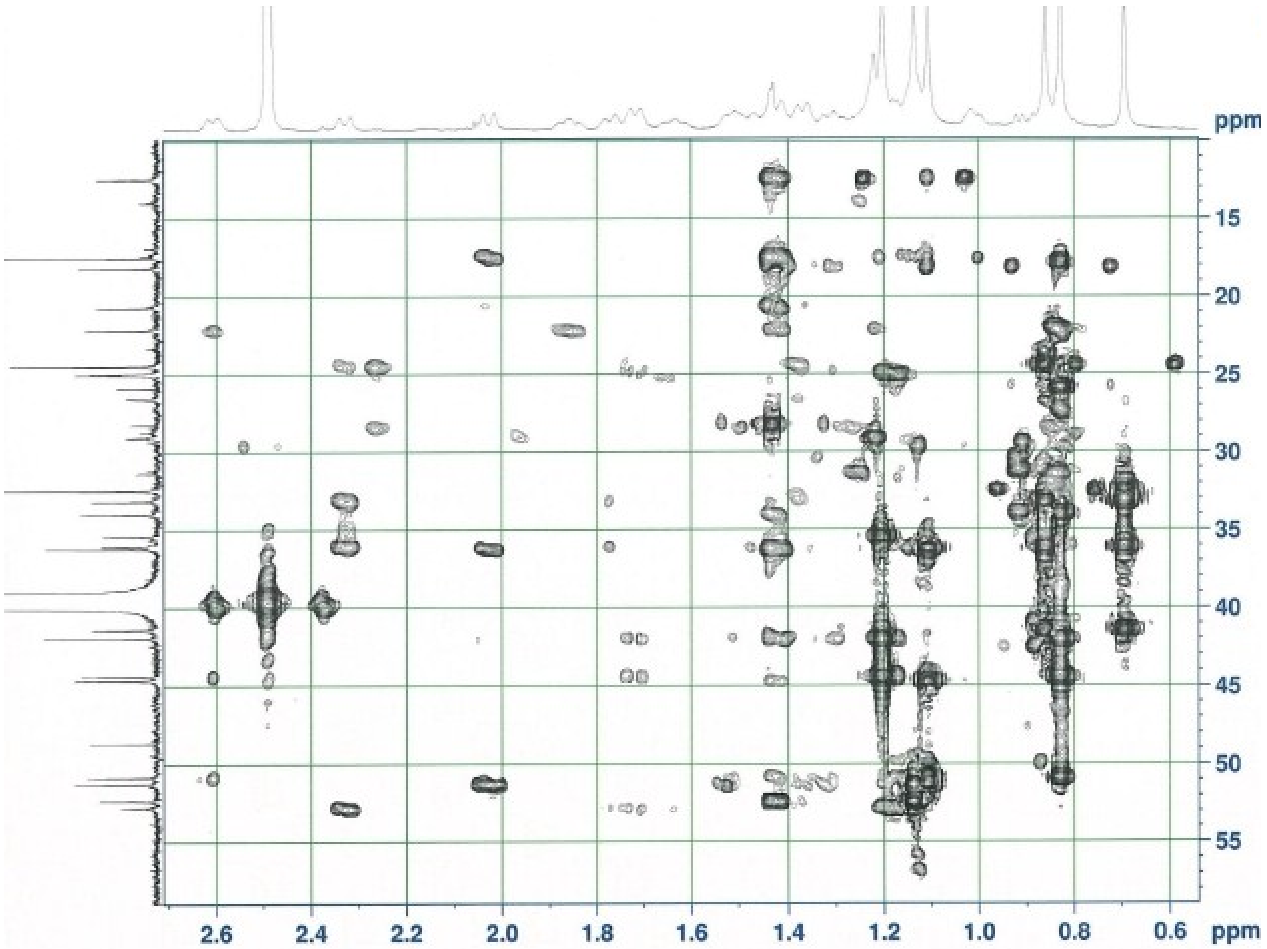

Fig. 28S. Expanded HMBC spectrum of compound 2

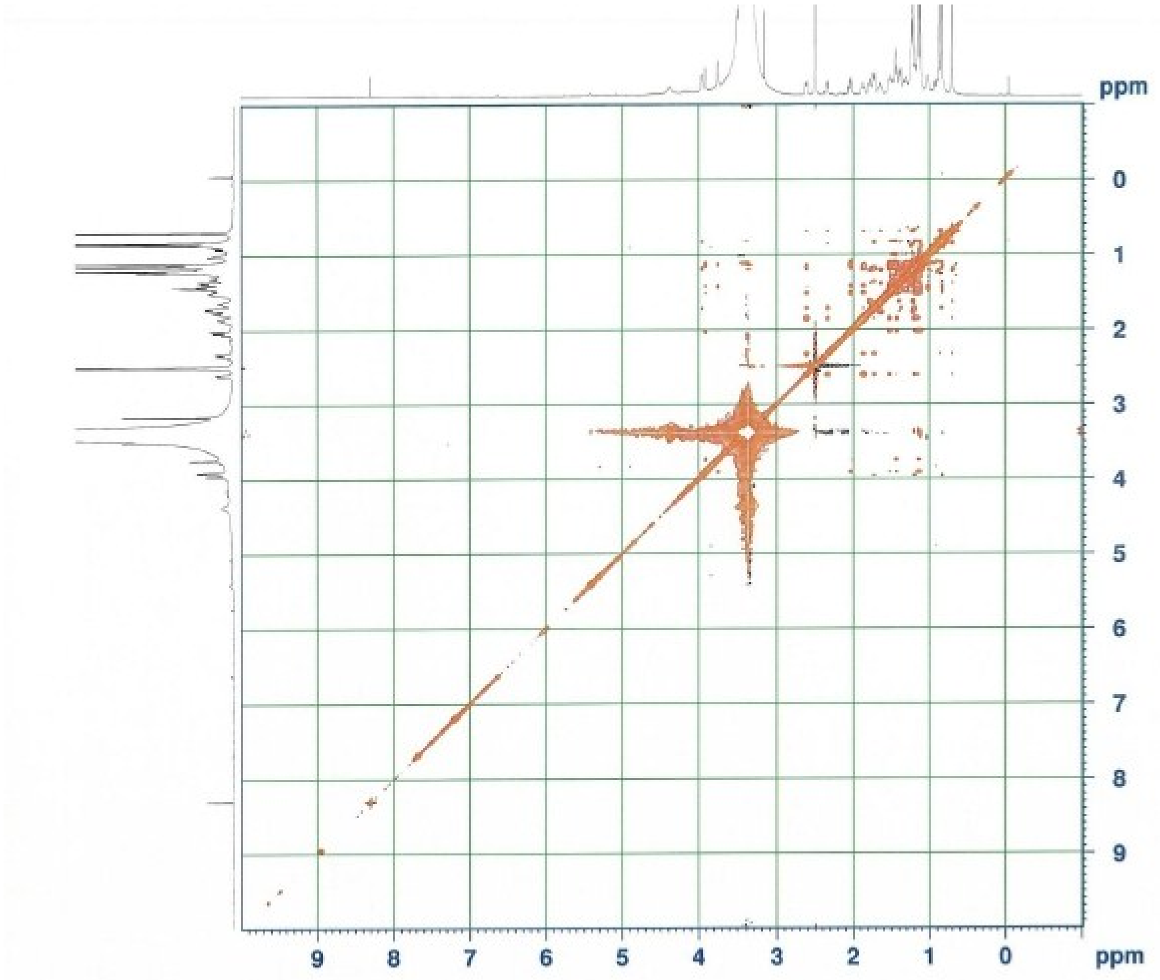

Fig. 29S. NOESY spectrum of compound 2

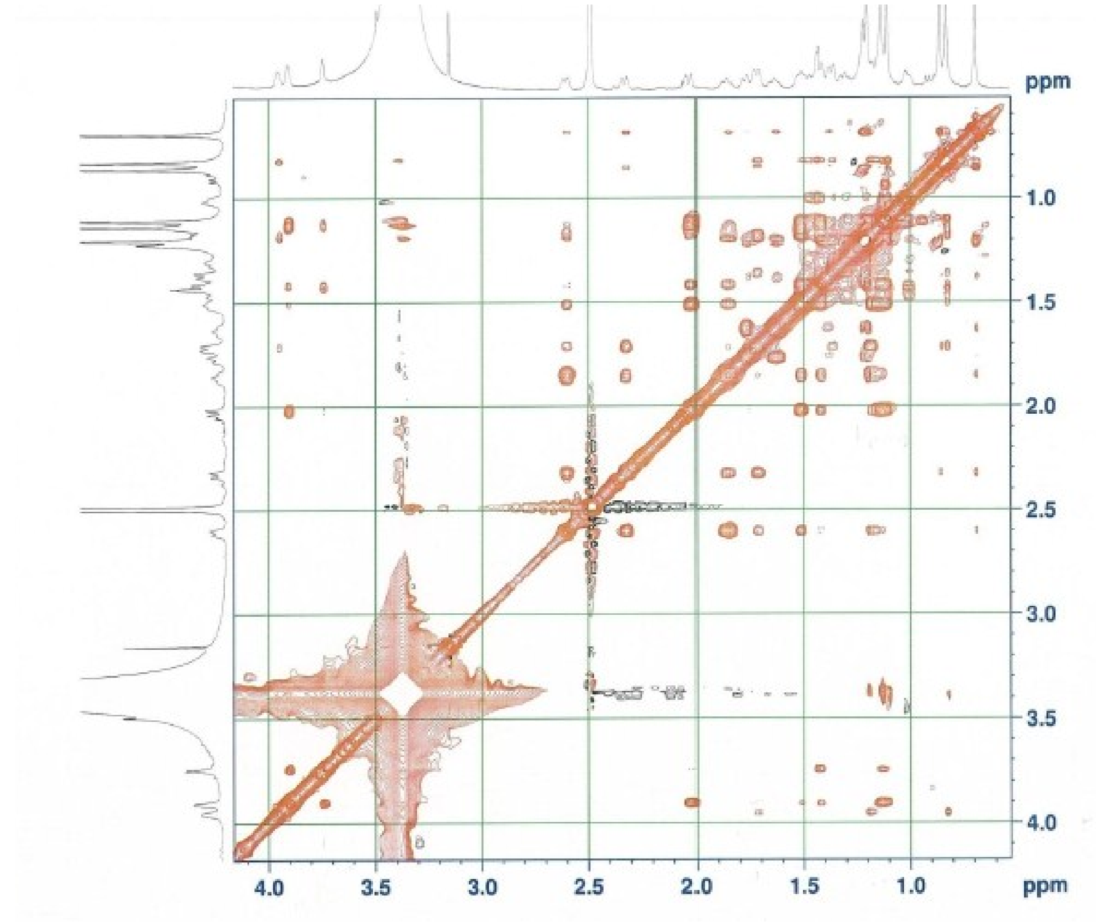

Fig. 30S. Expanded NOESY spectrum of compound 2

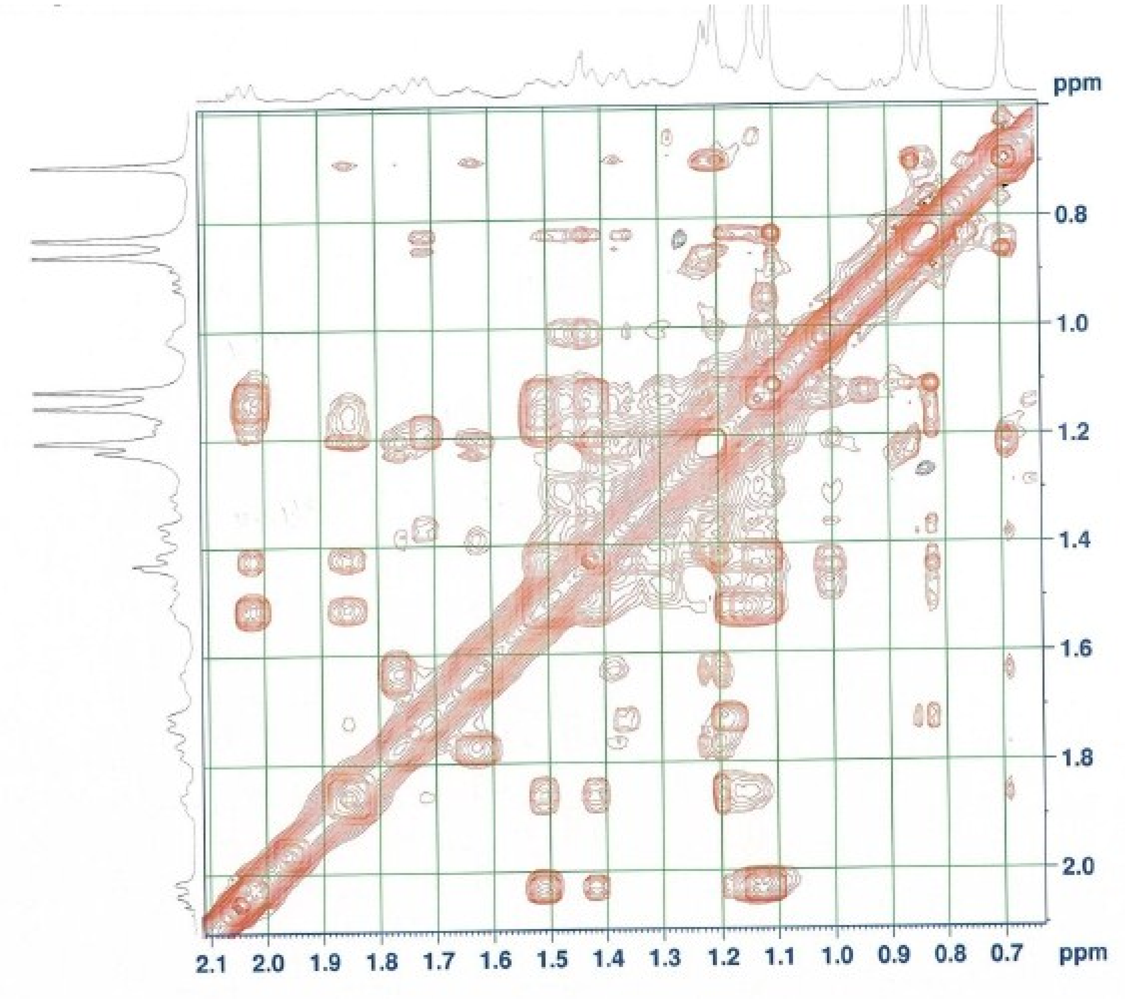

Fig. 31S. Expanded NOESY spectrum of compound 2

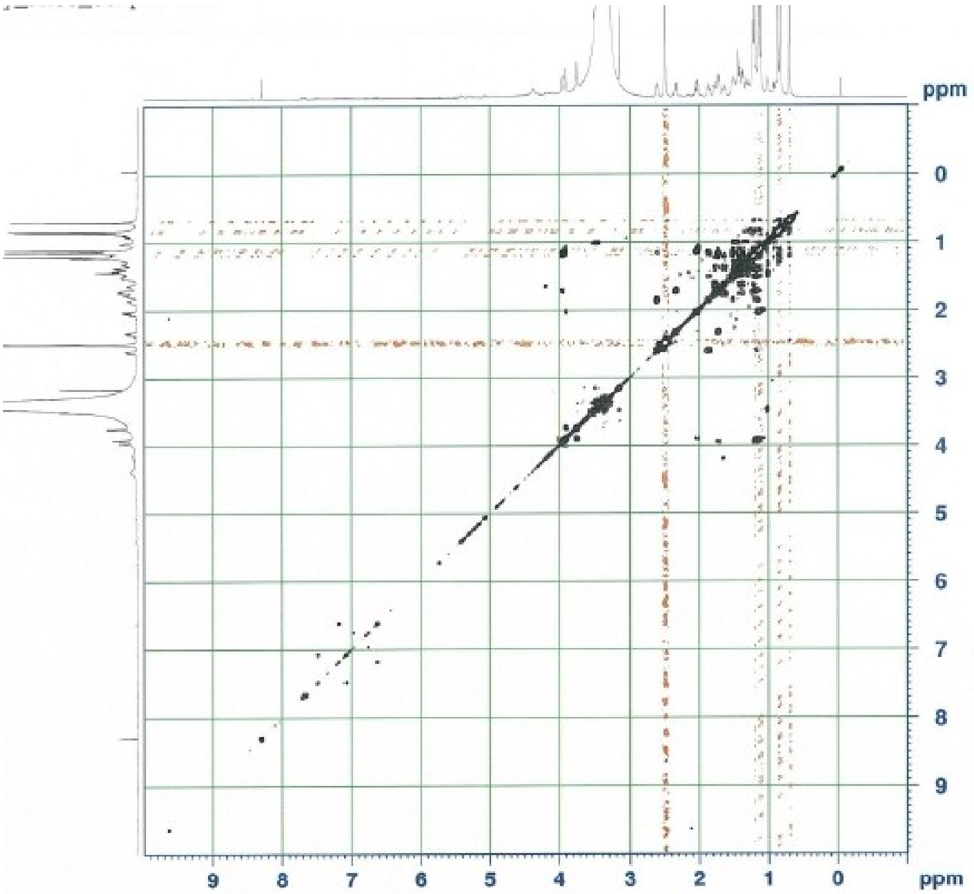

Fig. 32S. ^{1}H-^{1}H COSY spectrum of compound 2

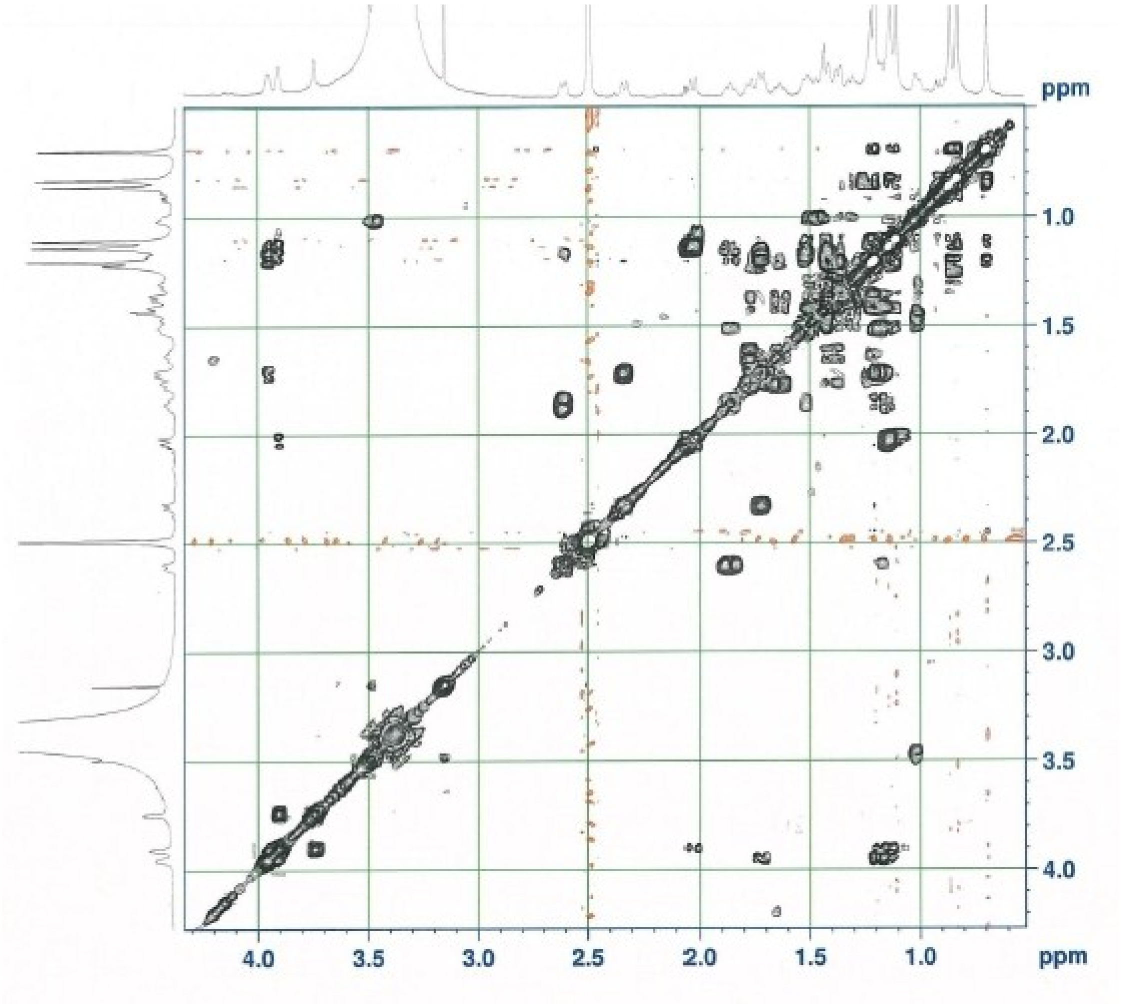

Fig. 33S. Expanded ^{1}H-^{1}H COSY spectrum of compound 2

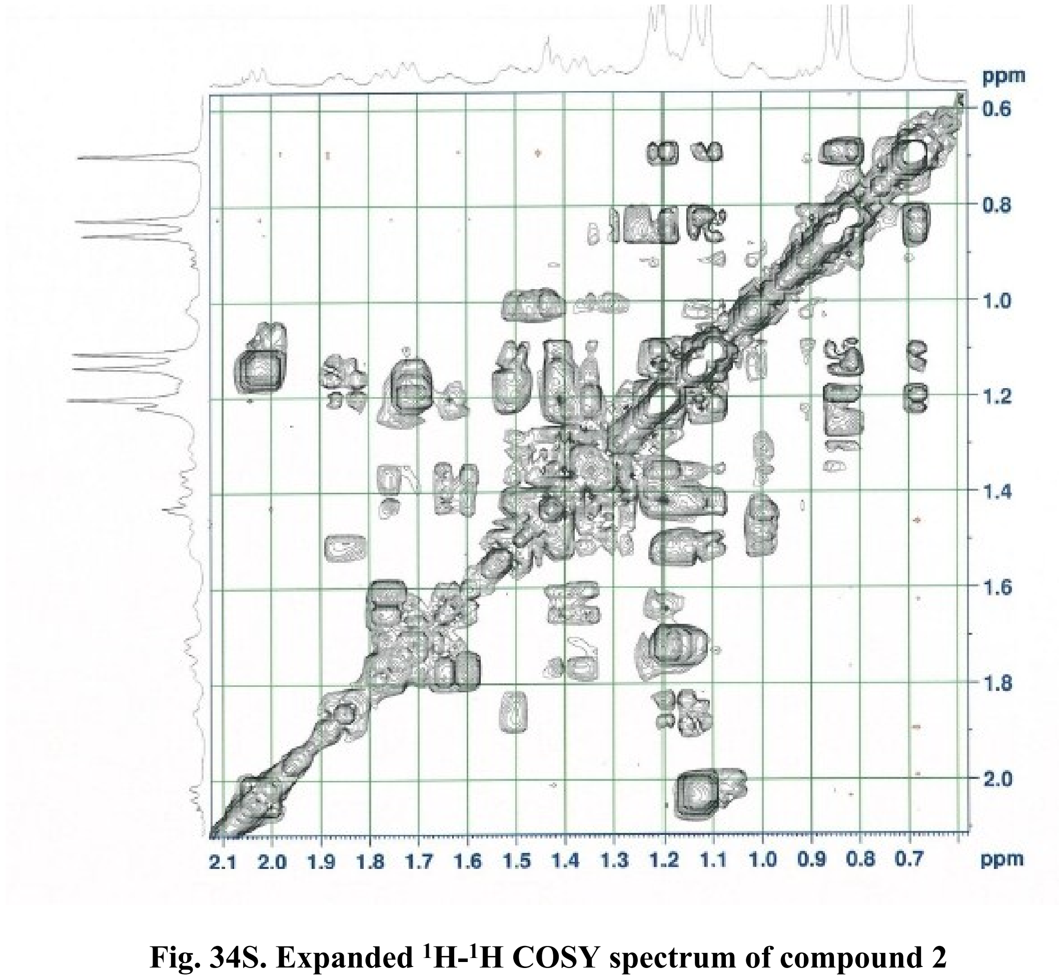

Fig. 34S. Expanded ^{1}H-^{1}H COSY spectrum of compound 2

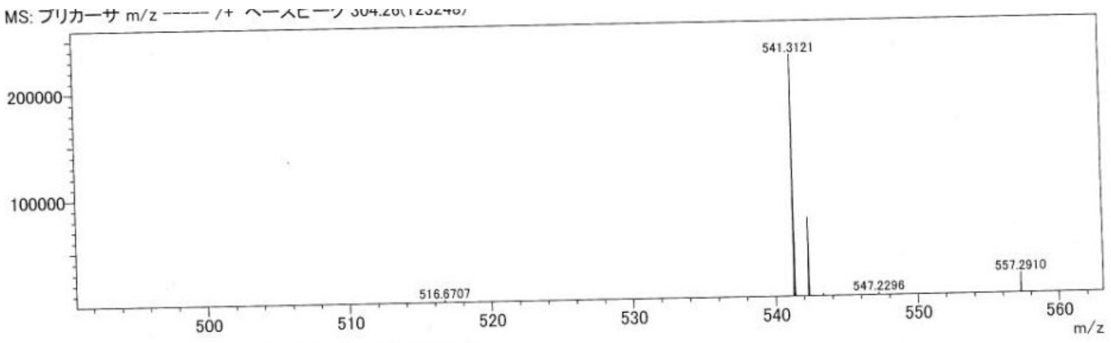

Fig. 35S. HR-ESI-MS spectrum of compound 2

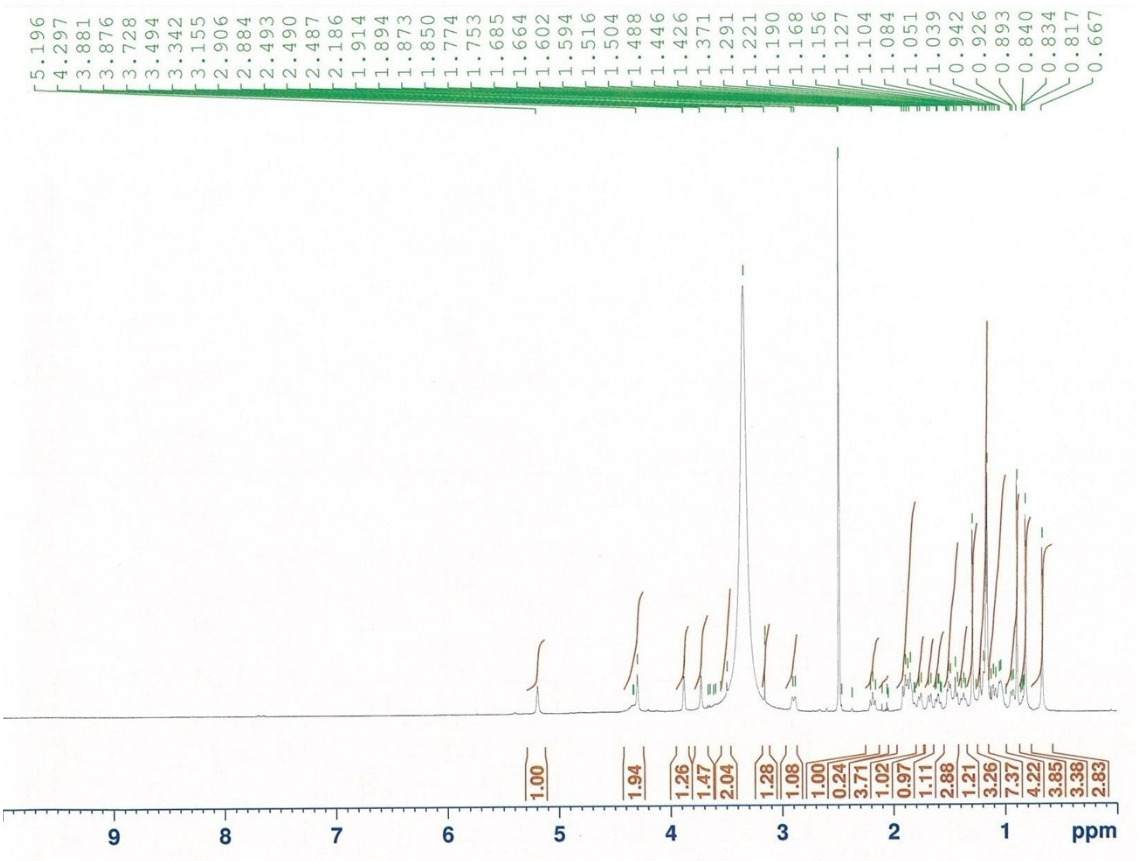

Fig. 36S. ^{1}H-NMR spectrum of compound 3 (600 MHz, DMSO-d_6)

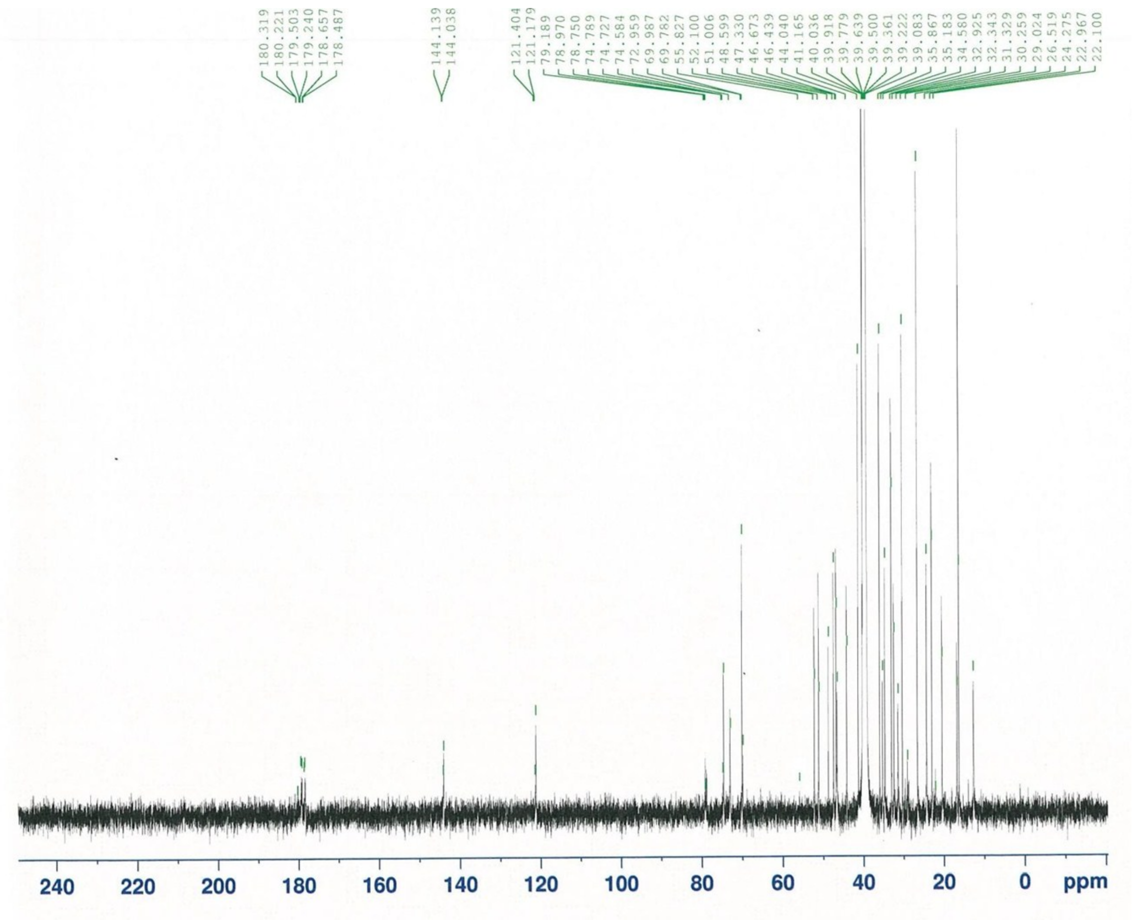

Fig. 37S. ^{13}C-NMR spectrum of compound 3 (150 MHz, DMSO-d_6)

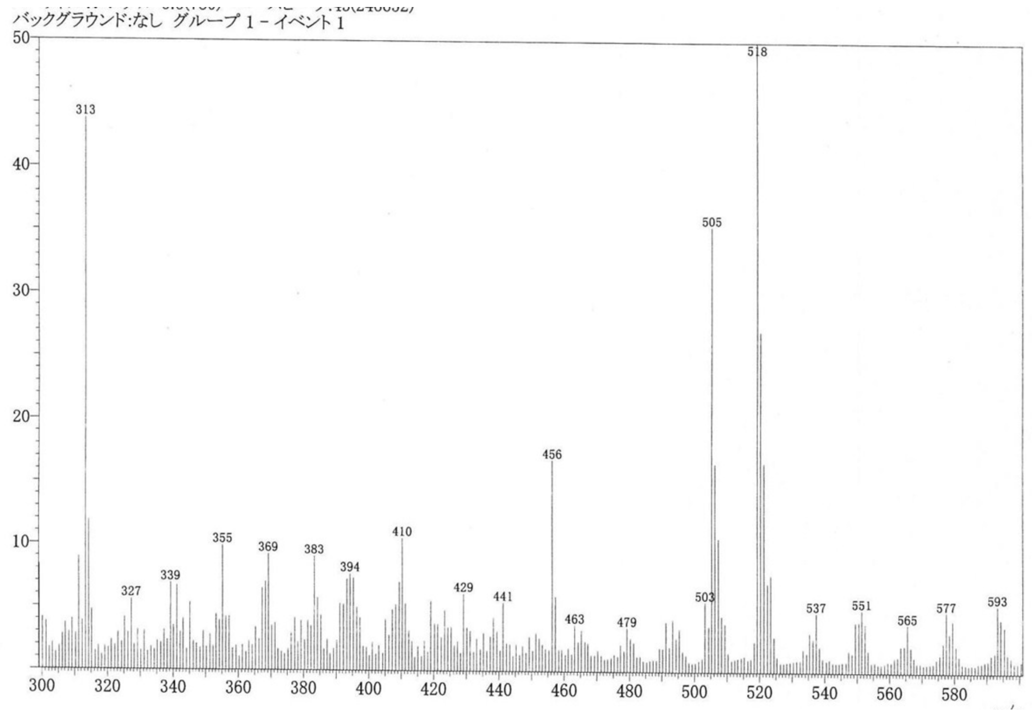

Fig. 38S. GC-MS spectrum of compound 3

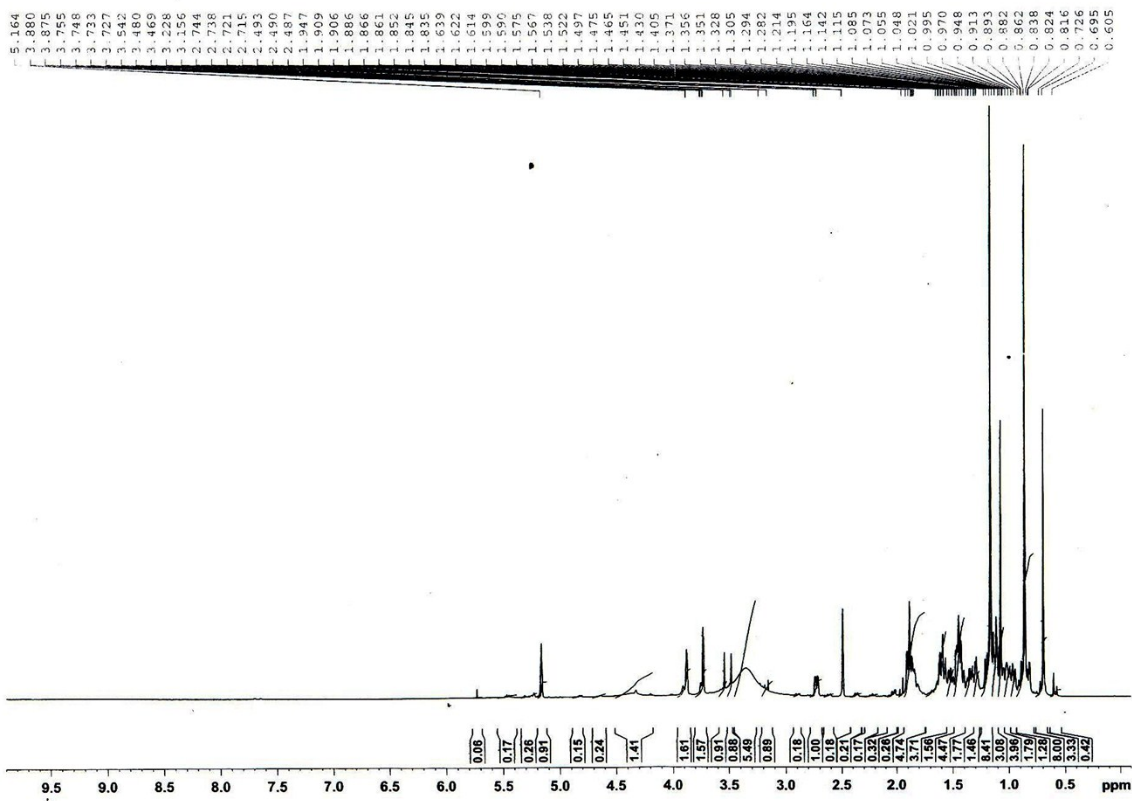

Fig. 39S. ^{1}H-NMR spectrum of compound 4 (600 MHz, DMSO-d_6)

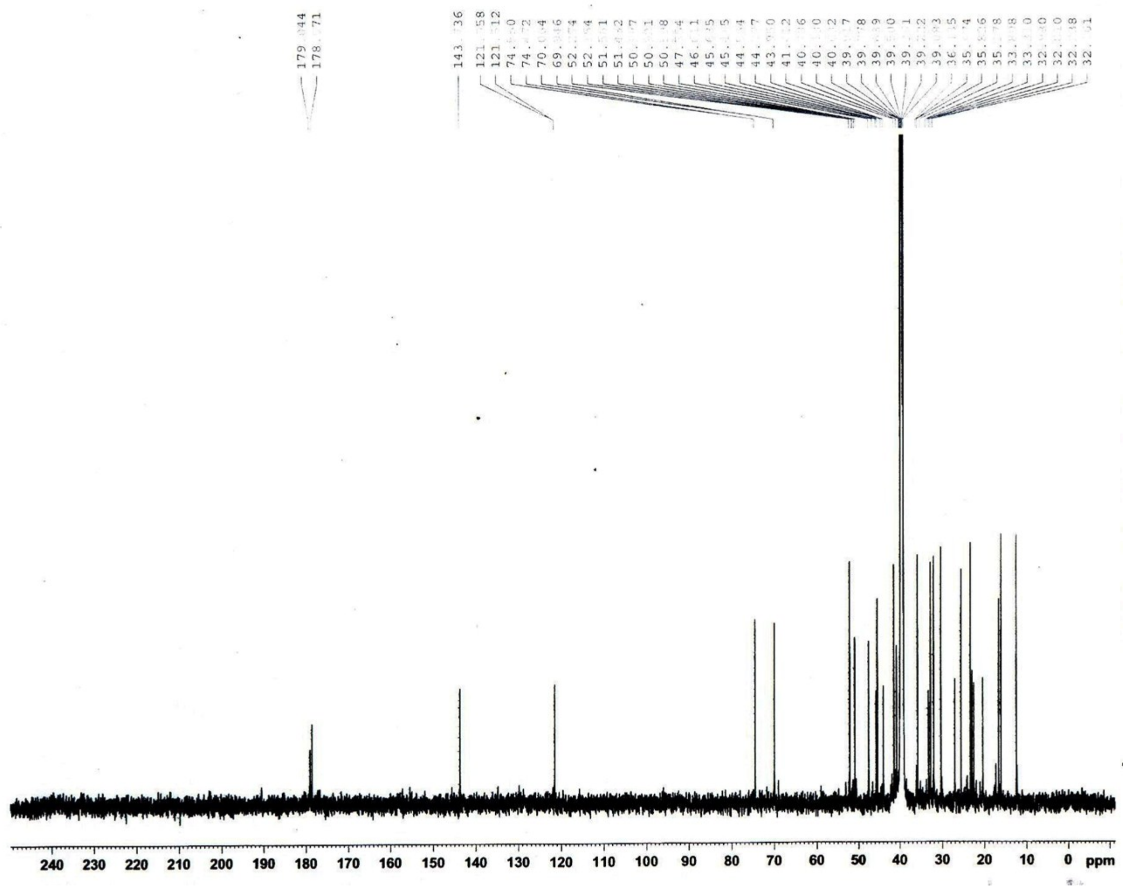

Fig. 40S. ^{13}C-NMR spectrum of compound 4 (150 MHz, DMSO-d_6)

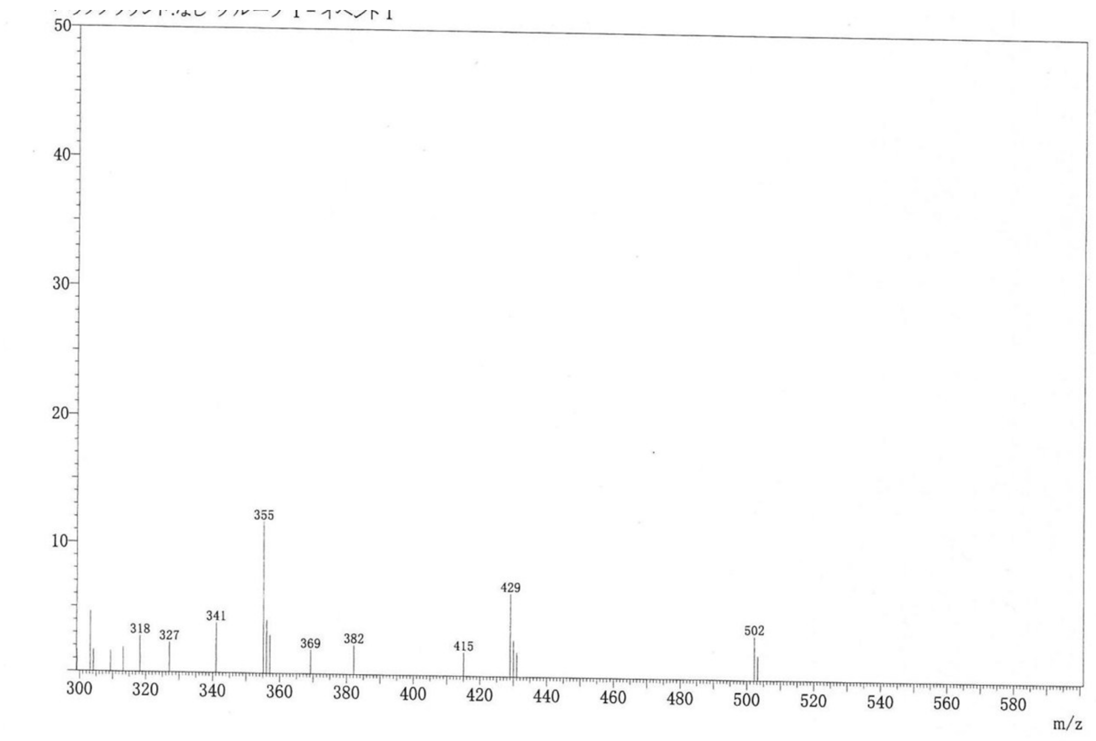

Fig. 41S. GC-MS spectrum of compound 4
